# Supplementary material for: Dental morphology in Homo habilis and its implications for the evolution of early Homo
Source: Nat Commun. 2024 Jan 4;15:286. doi: 10.1038/s41467-023-44375-9 (PMC10767101; doi:10.1038/s41467-023-44375-9)
Supplement: Supplementary file 1 — Supplementary Information [file 41467_2023_44375_MOESM1_ESM.pdf]

Supplementary Information

Dental morphology in *Homo habilis* and its implications for the evolution of early *Homo*

Thomas W. Davies*[a,b], Philipp Gunz[a], Fred Spoor[a,c], Zeresenay Alemseged[d], Agness Gidna[e], Jean-Jacques Hublin[f,g], William H. Kimbel[h†], Ottmar Kullmer[i,j], William P. Plummer[b], Clément Zanolli[k], Matthew M. Skinner[b,l]

a - Department of Human Origins, Max Planck Institute for Evolutionary Anthropology, Leipzig, Germany

b - School of Anthropology and Conservation, University of Kent, Canterbury, United Kingdom

c - Centre for Human Evolution Research, Natural History Museum, London, United Kingdom

d - Department of Organismal Biology and Anatomy, University of Chicago, Chicago, Illinois, United States

e - Department of Cultural Heritage, Ngorongoro Conservation Area Authority, P. O. Box 1, Ngorongoro Crater, Arusha, Tanzania

f - Collège de France, Paris, France

g - Max Planck Institute for Evolutionary Anthropology, Leipzig, Germany

h - Institute of Human Origins, and School of Human Evolution and Social Change, Arizona State University, Tempe, Arizona, United States

i - Palaeobiology and Environment workgroup, Institute of Ecology, Evolution, and Diversity, Goethe University, Frankfurt, Germany

j - Division of Palaeoanthropology, Senckenberg Research Institute and Natural History Museum Frankfurt, Frankfurt am Main, Germany

k - Univ. Bordeaux, CNRS, MCC, PACEA, UMR 5199, F-33600 Pessac, France

l - Centre for the Exploration of the Deep Human Journey, University of the Witwatersrand, Johannesburg, South Africa

* Corresponding author

Email: thomas_davies@eva.mpg.de

Supplementary Notes

Supplementary Note 1 – *Homo habilis* derived dental traits.

There are several dental traits that have been suggested to be derived for *Homo habilis* relative to *Australopithecus*, such as buccolingually narrow tooth crowns. In the initial description of the species, all tooth crowns were found to be buccolingually narrow compared to the *Australopithecus* sample known at the time, but it was found that this pattern was clearest in the lower premolars[1]. This shape difference was also emphasised in Tobias[2,3] and Wood[4]. Some subsequent analyses of buccolingual narrowing have found that it is limited only to some tooth positions; in the mandibular dentition, White et al.[5] and Suwa et al.[6] found that only the *Homo* M_1s were buccolingually narrow, however in these cases a mixed early *Homo* sample was used that included specimens attributed to *H. habilis*, *Homo rudolfensis* and *Homo erectus*. Similarly, in the maxillary teeth White et al.[5] found that only early *Homo* P^4s and M^1s were narrow compared to *Australopithecus*. Further, Kimbel et al.[7] found that the pattern of buccolingual narrowing in *H. habilis* was clearest in the M^1s.

Using published and original buccolingual and mesiodistal linear measurements of P^3-M^3 and P_4-M_3 [4,7–17], we calculated shape indices for all currently published specimens of *Australopithecus afarensis*, *Australopithecus africanus*, and *H. habilis* (Supplementary Table 1), as well as for a subsample of these hypodigms that matches the specimens included in the EDJ analysis presented here (Supplementary Table 2; specimens included in this subsample are listed in Supplementary Table 4, indicated by 'EDJ' column). Using t-tests to compare *H. habilis* with a pooled *Australopithecus* sample for each tooth position, we find that P_4-M_3 in *H. habilis* are narrower than *Australopithecus* sample. For the maxillary teeth, the P^3 and M^1 are narrower in *H. habilis* than *Australopithecus*, while the P^4s are narrower only in the full sample (no significant difference was found in the EDJ matched subsample, which is likely due to the smaller sample sizes). Thus, many tooth positions do, indeed, show significant narrowing of the crown in *H. habilis* compared with *Australopithecus* when assessed at the outer enamel surface (OES).

At the enamel-dentine junction, we find that some *H. habilis* teeth are relatively narrower buccolingually than *Australopithecus*, but this varies by specimen, tooth position and which part of the EDJ is being considered. For example, wireframe models of mean landmark positions (Supplementary Figure 1) show that the *H. habilis* M_1 CEJ (representing the shape of the base of the crown) is relatively narrower buccolingually than that of *Australopithecus*, but this is not the case in the M_2s. However, when considering the shape of the EDJ marginal ridges (Supplementary Figure 2), both M_1 and M_2 have mean shapes that are very similar to *Australopithecus*. OH 7 and KNM-ER 1802 show narrowing in M_1s and M_2s at both CEJ ridge and EDJ ridge level, while OH 13 shows no narrowing in M_1 or M_2, and a OH 16 only shows narrowing in the M_1, not M_2.

Overall, the buccolingual narrowing observed at the OES is far less clear at the EDJ. In some cases, such as the M^1, the narrowing observed in OES linear dimensions may be obscured by the different crown shape in *H. habilis*, which is expanded on the mesiobuccal side of the CEJ, when compared to *Australopithecus*, which is expanded on the mesiodistal side. This shape difference is not well-characterised as buccolingual narrowing at the CEJ, but it could contribute to a narrower shape index as measured through linear OES dimensions. It is also important to

consider differences in the distribution and relative thickness of enamel over the crown; relatively thicker enamel on the mesial and/or distal faces of the tooth in *Homo* could extend the mesiodistal dimensions of the tooth. Enamel thickness is often measured in 2D in teeth that are naturally fractured [18,19], physically sectioned [20,21], or virtually sectioned [22–26]. However, questions of enamel thickness distribution require methods that measure enamel thickness in 3D [27–31]. These 3D enamel thickness methods have so far been applied to only a small number of *Australopithecus* and early *Homo* specimens [29,30,32]; applying these methods more widely may help address this issue.

Another suggested distinctive *H. habilis* trait is large anterior teeth. Leakey et al.[1] found the incisors are large compared to *Australopithecus* and *H. erectus*, and that the canines are proportionally large relative to the premolars. In our analysis of crown base (CEJ) size, we find that OH 16 has relatively large anterior teeth, particularly the mandibular incisors, which are larger than most specimens of *Australopithecus* or *H. erectus* included here. The OH 15 canine is also particularly large. However, the anterior teeth of OH 7 are within the range of *Australopithecus*, while the preserved anterior teeth of OH 13 (C_1), OH 39 (I^1, C^1) and KNM-ER 1813 (I^2, C^1) are all small relative to *Australopithecus*. Further, we found no significant differences between the anterior teeth of *H. habilis* and *Australopithecus* in permutation tests of centroid size (Supplementary Table 3), although the sample sizes for *H. habilis* are small. This method of quantifying crown size does not consider enamel thickness, which may contribute to the patterns observed at the OES.

Supplementary Figures

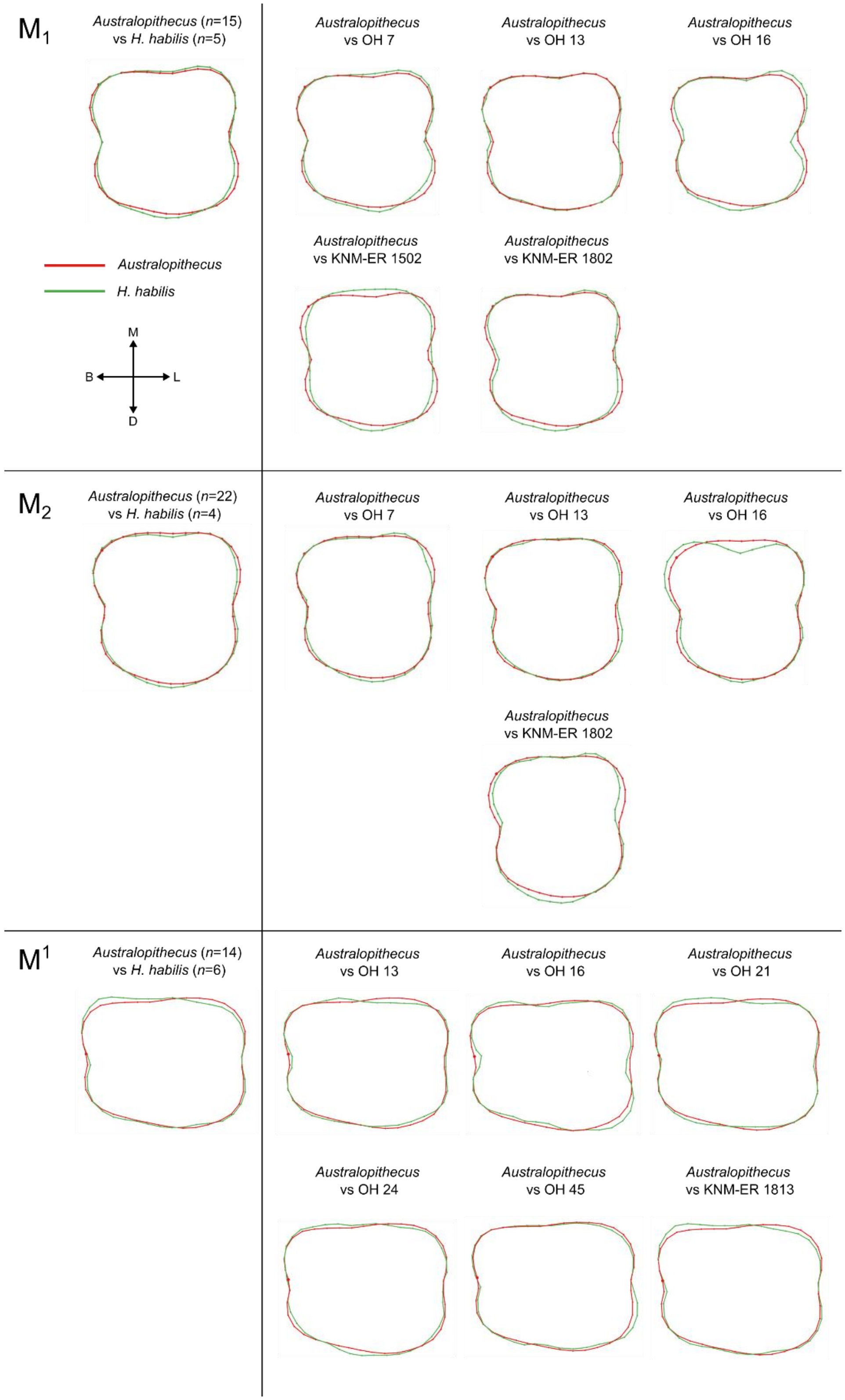

Supplementary Figure 1 – Wireframe images of the CEJ shape differences between *Australopithecus* and *Homo habilis*. Images show the mean *Australopithecus* CEJ ridge landmark configuration versus the *Homo habilis* mean (left), and versus each *H. habilis* specimen separately (right), for the M_1, M_2 and M^1. All are shown in occlusal view. *n* = number of teeth used to generate each mean model

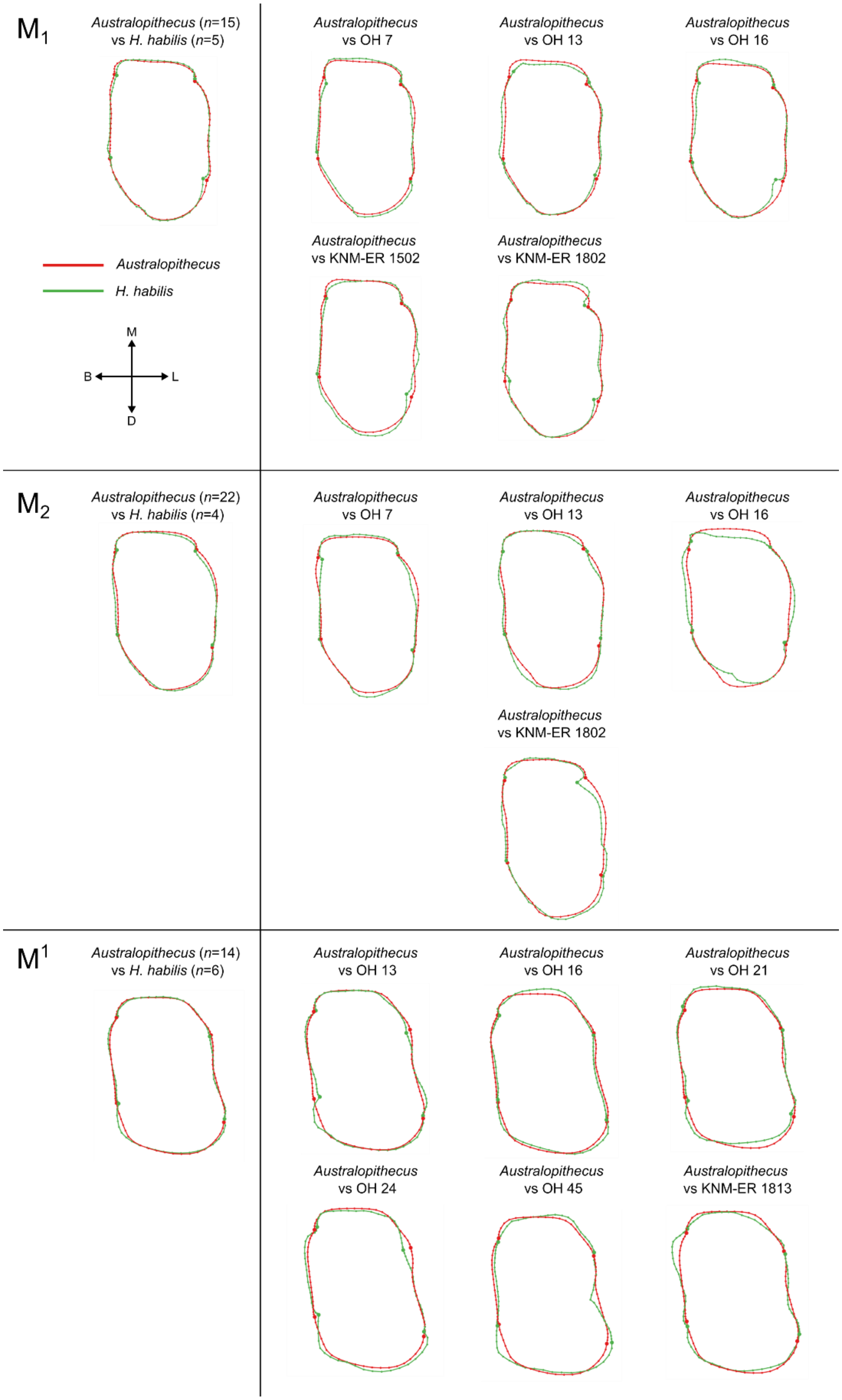

Supplementary Figure 2 – Wireframe images of the EDJ shape differences between *Australopithecus* and *Homo habilis*. Images show the mean *Australopithecus* EDJ ridge landmark configuration versus the *Homo habilis* mean (left), and versus each *H. habilis* specimen separately (right), for the M_1, M_2 and M^1. All are shown in occlusal view. *n* = number of teeth used to generate each mean model

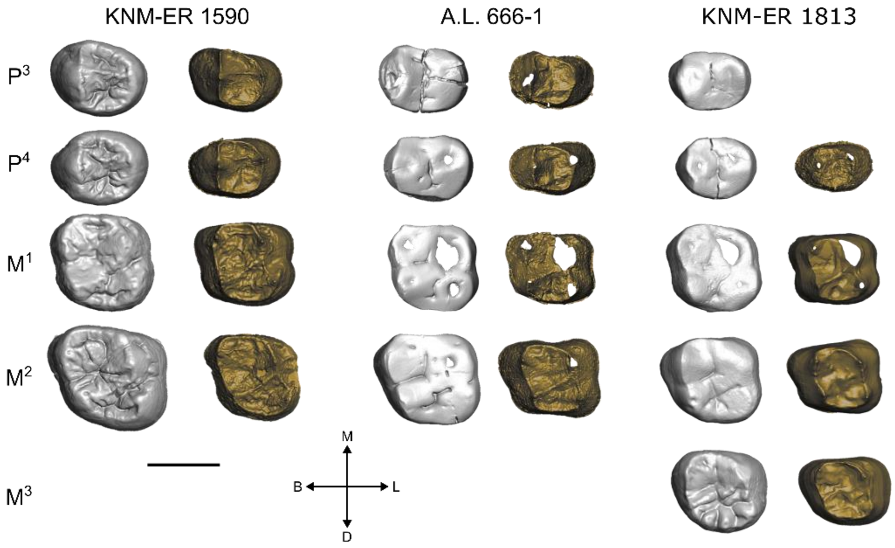

Supplementary Figure 3 – Koobi Fora and Hadar maxillary tooth rows: KNM-ER 1590 (*Homo* sp.), A.L. 666-1 (*Homo* aff. *H. habilis*), and KNM-ER 1813 (*H. habilis*). Each is shown in occlusal view at the outer enamel surface (left) and enamel-dentine junction (right). All teeth are shown as right sided.

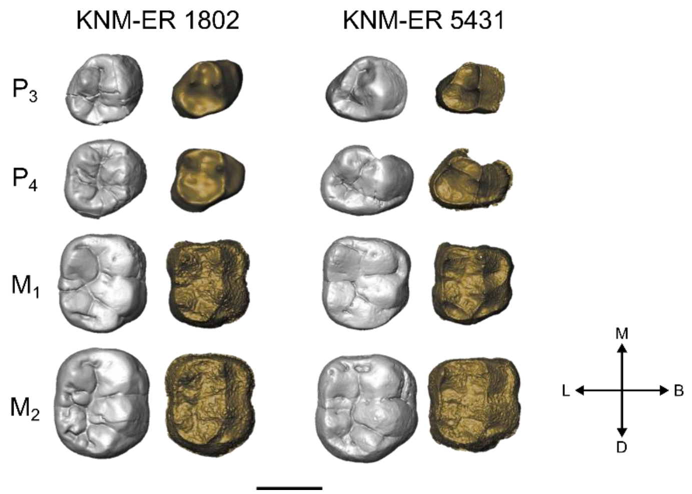

Supplementary Figure 4 – Koobi Fora mandibular tooth rows: KNM-ER 1802 (*H. habilis*) and KNM-ER 5431 (Indet). Both are shown in occlusal view at the outer enamel surface (left) and enamel-dentine junction (right). All teeth are shown as right sided

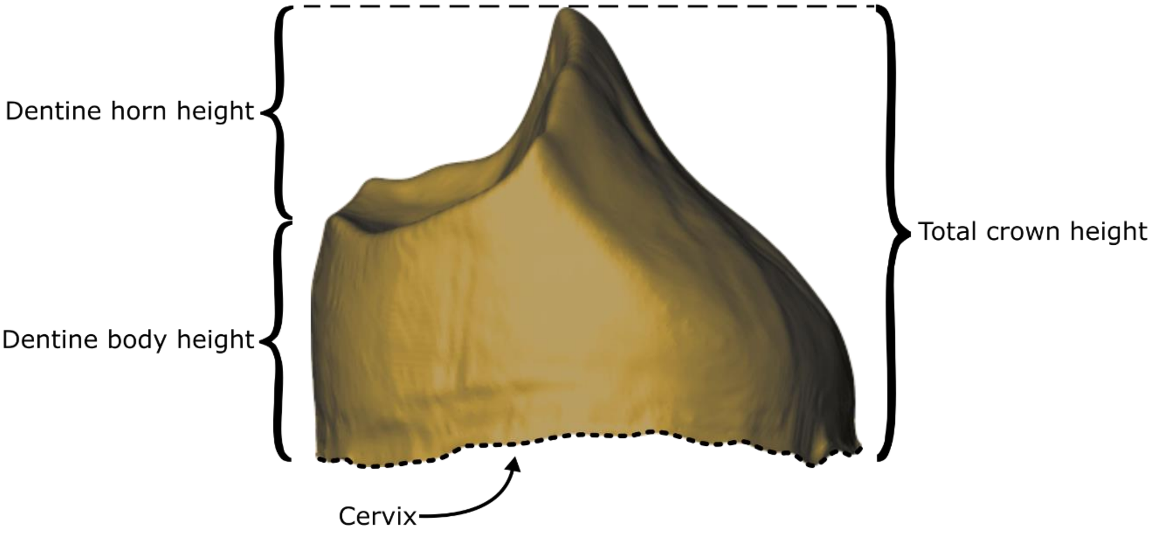

Supplementary Figure 5 – Example premolar illustrating the terms used to describe crown height at the enamel-dentine junction

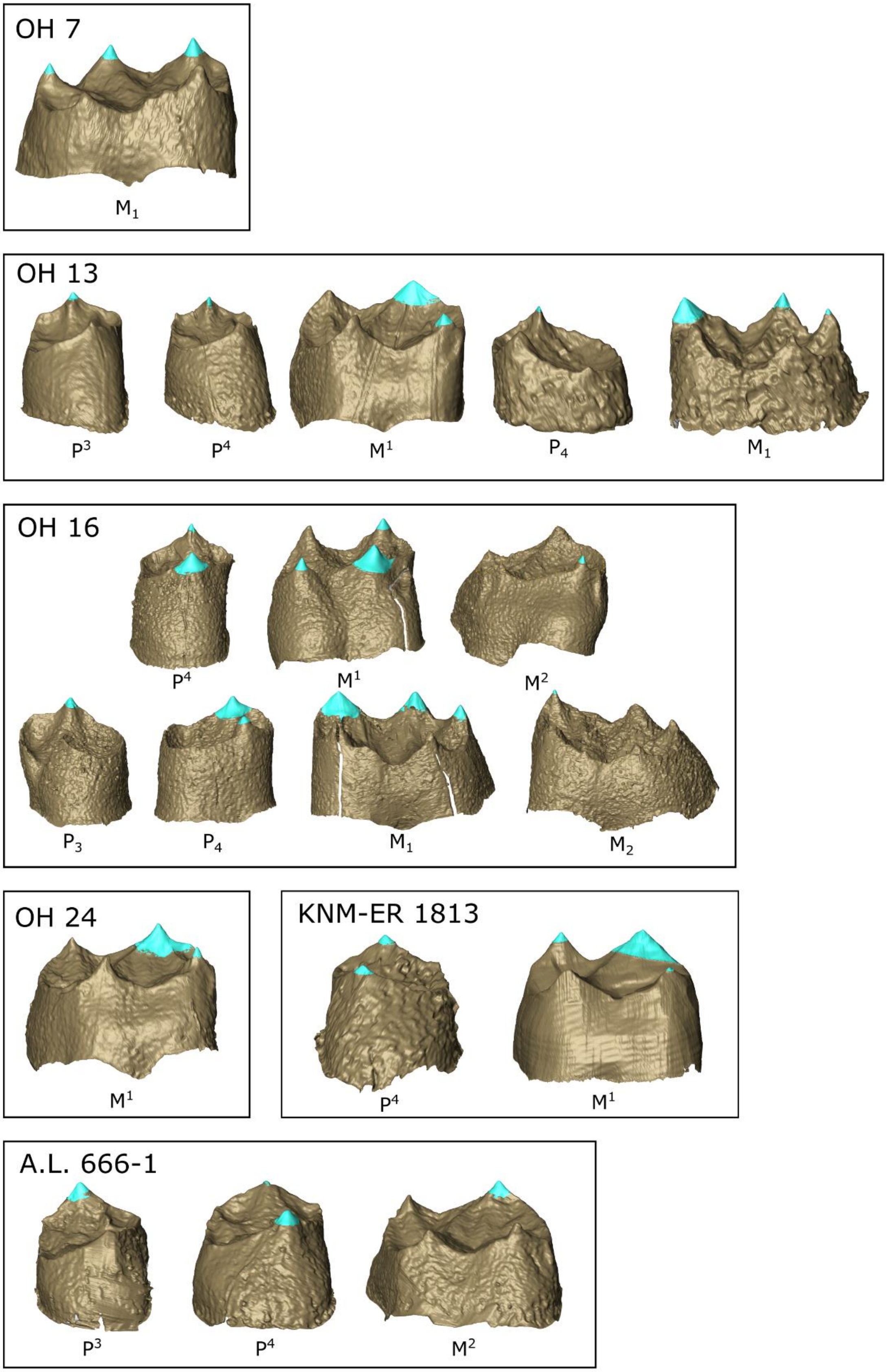

Supplementary Figure 6 – Images of cusp reconstructions in *H. habilis* specimens. Each *H. habilis* specimen with reconstructed dentine horns is shown, with the reconstructed region shown in blue.

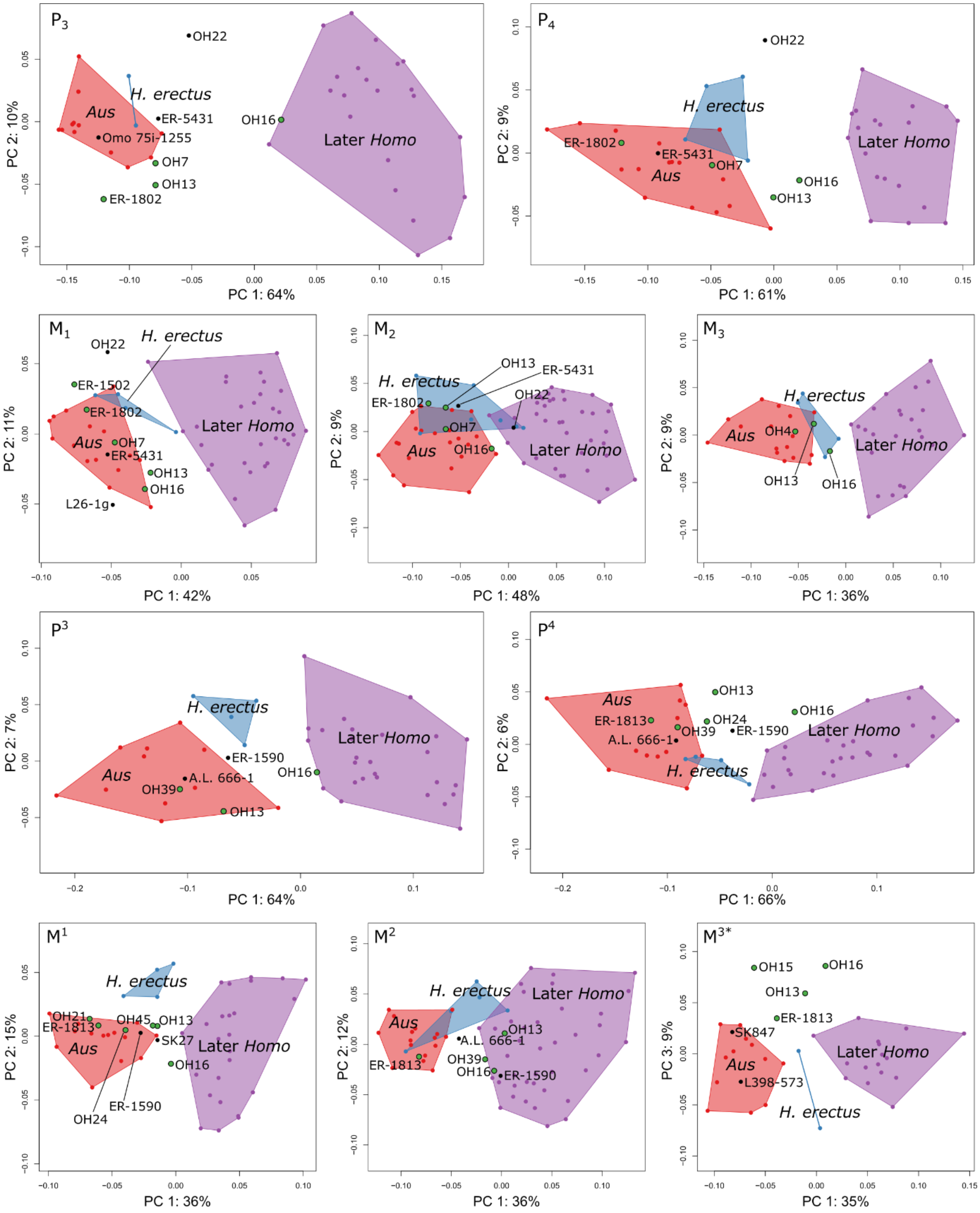

Supplementary Figure 7 – PCA plots showing variation in EDJ shape for each postcanine tooth position. *Homo habilis* specimens are represented by green points. All plots show the first two principal components, except those marked with an asterisk (*), which show the first and third principal components. *Aus* = *Australopithecus*. Source data are provided as a Source Data file.

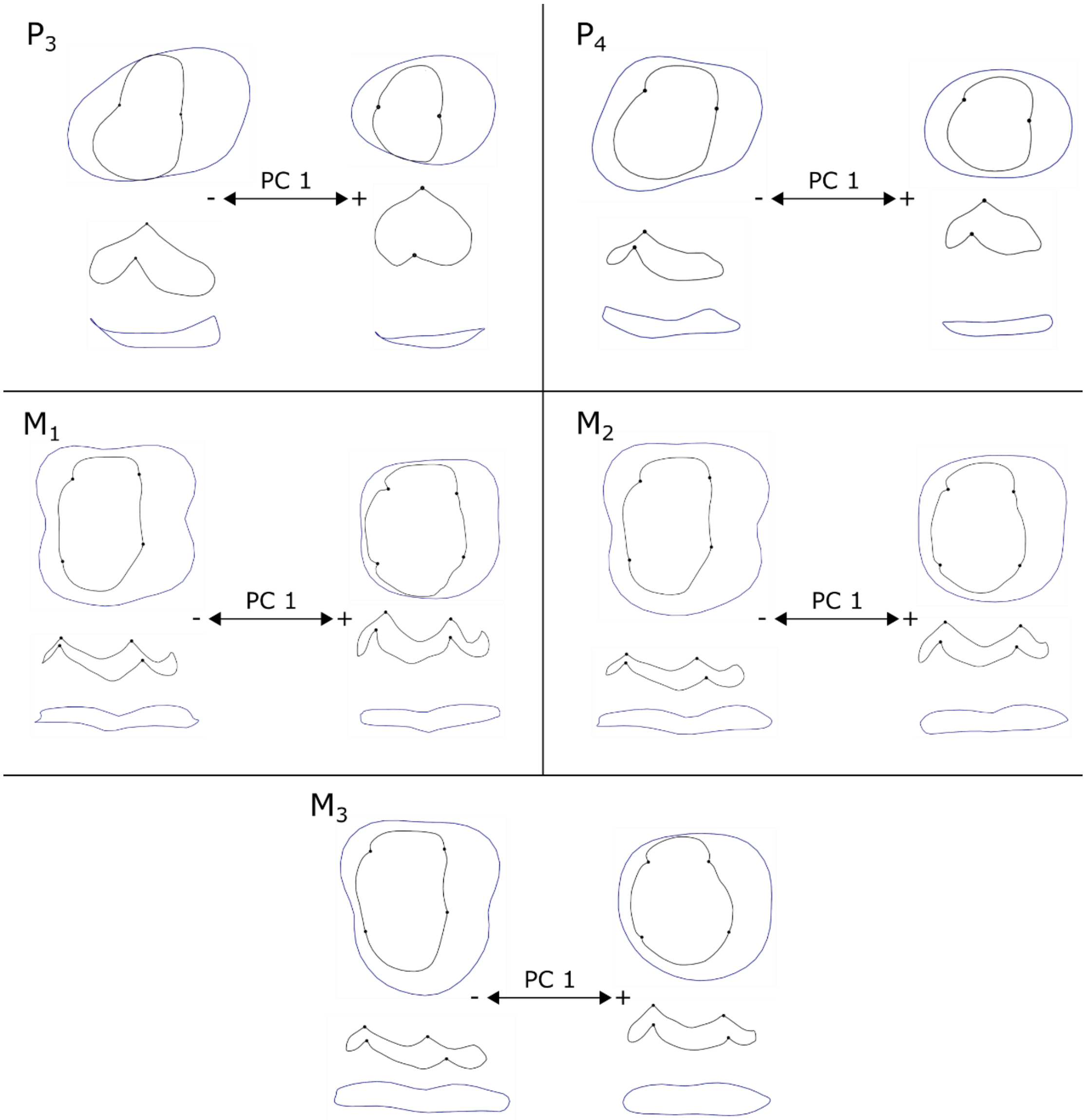

Supplementary Figure 8 – Wireframe images of the shape changes associated with the first principal component for each mandibular tooth position. For each tooth, the wireframes show the landmark positions associated with the extremes of principal component 1 (PC1) (± 1.5 standard deviations from the mean). Wireframes for each tooth position show CEJ ridge landmarks (blue lines), EDJ ridge landmarks (black lines) and landmarks for the main cusps (black circles) in occlusal view (top) and lingual view (bottom).

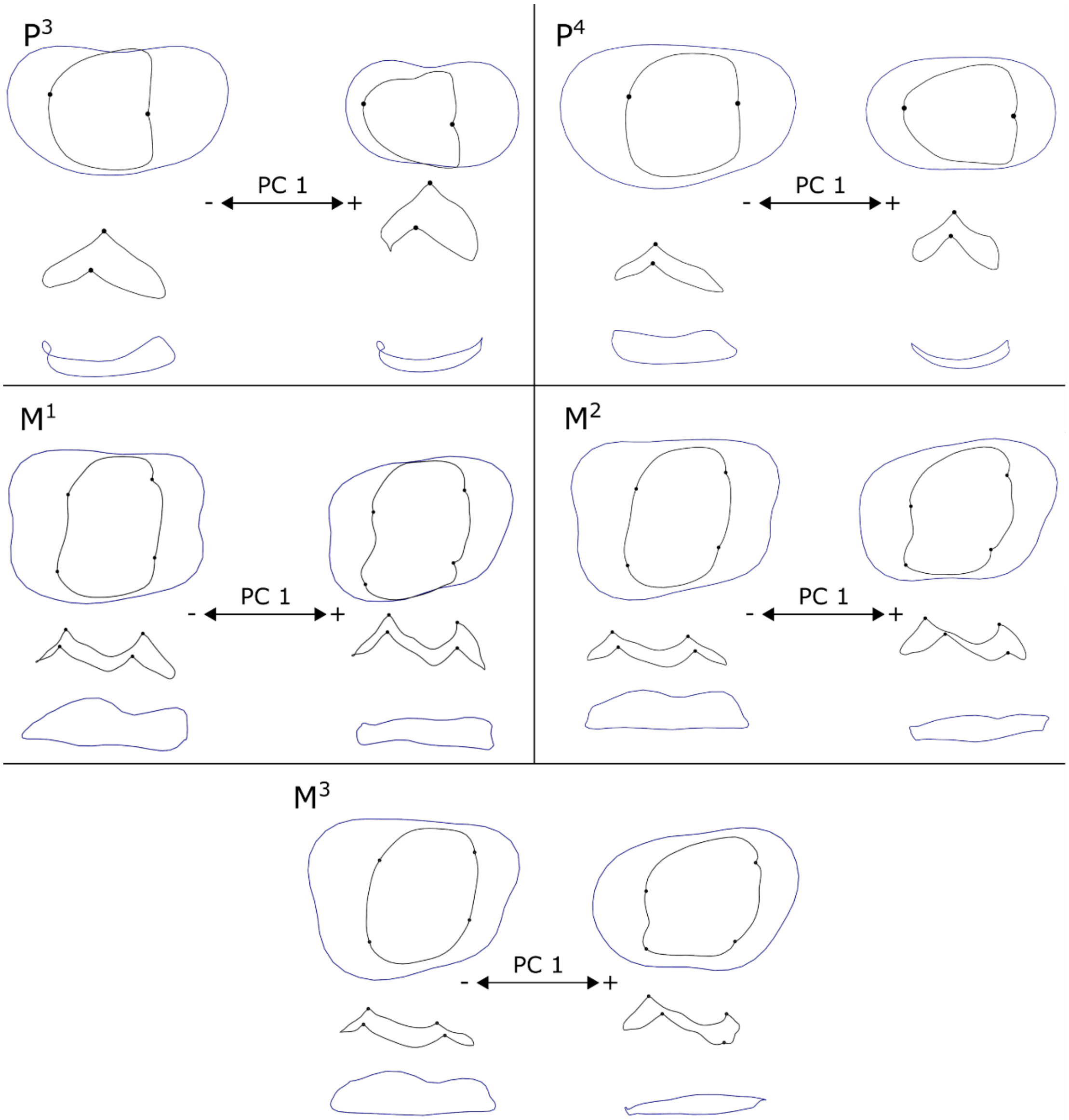

Supplementary Figure 9 – Wireframe images of the shape changes associated with the first principal component for each maxillary tooth position. For each tooth, the wireframes show the landmark positions associated with the extremes of principal component 1 (PC1) (± 1.5 standard deviations from the mean). Wireframes for each tooth position show CEJ ridge landmarks (blue lines), EDJ ridge landmarks (black lines) and landmarks for the main cusps (black circles) in occlusal view (top) and lingual view (bottom).

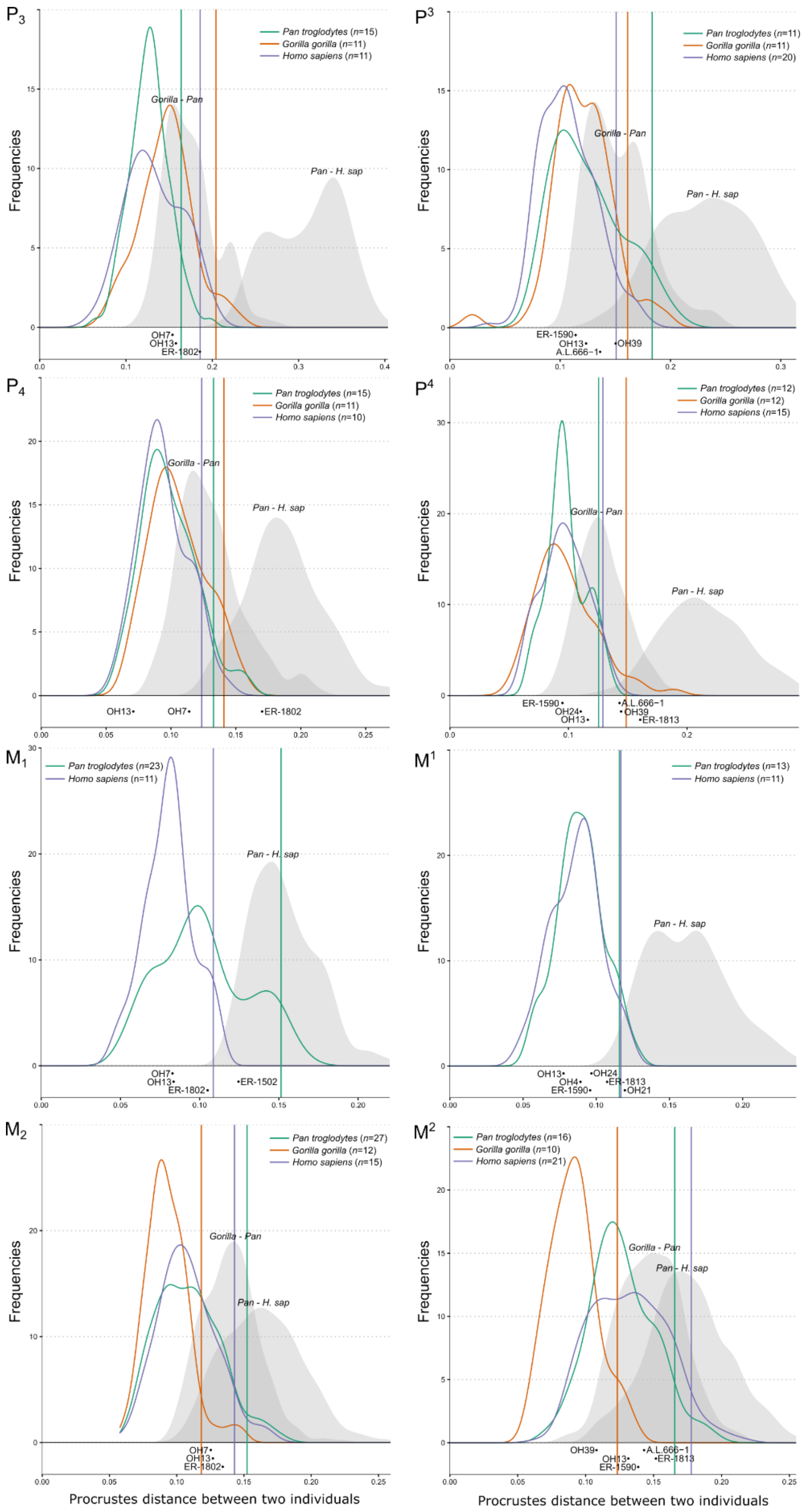

Supplementary Figure 10 – Frequency plots of Procrustes distances between all possible pairs of individuals within groups (shown by coloured lines) and between groups (grey) for each postcanine tooth position. Vertical lines show the 95% limits of the within group distributions. Below each plot is shown the Procrustes distance between OH 16 and other *Homo habilis* or other early *Homo* specimens. Source data are provided as a Source Data file. *H. sap = Homo sapiens*

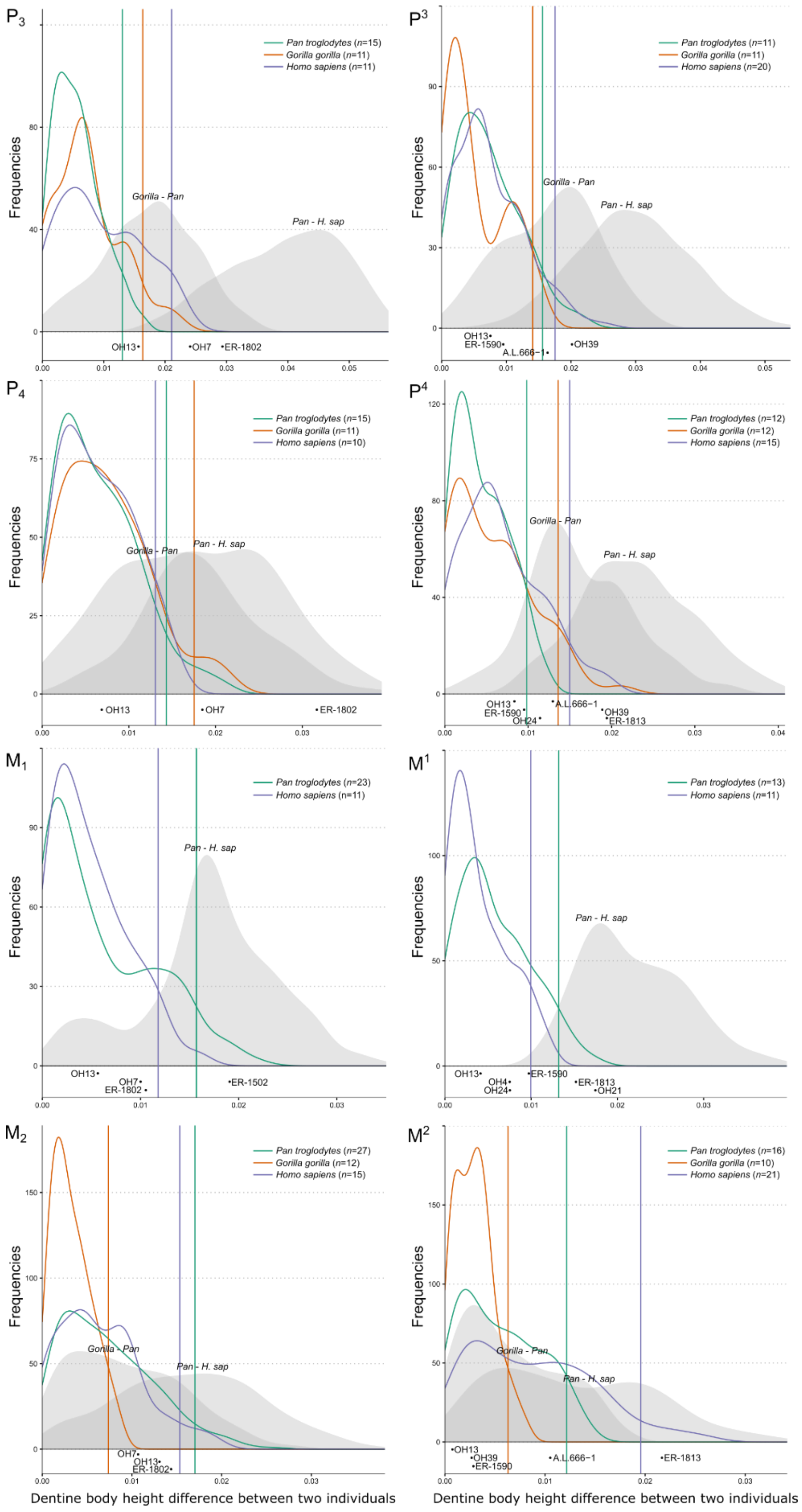

Supplementary Figure 11 – Frequency plots of difference in dentine body height between all possible pairs of individuals within groups (shown by coloured lines) and between groups (grey) for each postcanine tooth position. Vertical lines show the 95% limits of the within group distributions. Below each plot is shown the difference in dentine body height between OH 16 and other *Homo habilis* or other early *Homo* specimens. Source data are provided as a Source Data file. *H. sap* = *Homo sapiens*

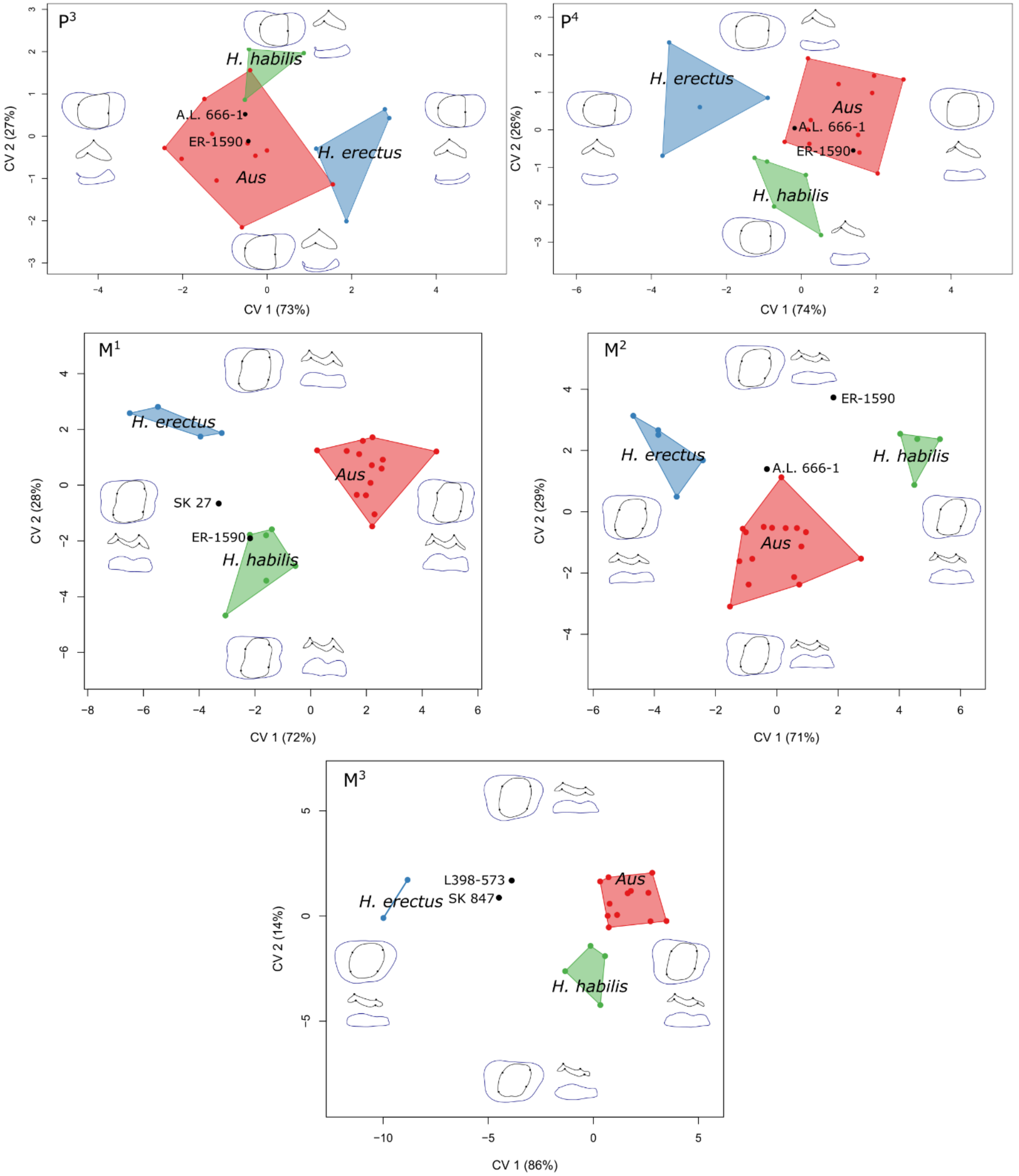

Supplementary Figure 12 – Canonical variate analysis (CVA) plots of EDJ shape in maxillary postcanine tooth positions. Wireframe images of CV (canonical variate) extremes are also shown (±1.5 standard deviations from the mean). *Aus* = *Australopithecus*. Source data are provided as a Source Data file.

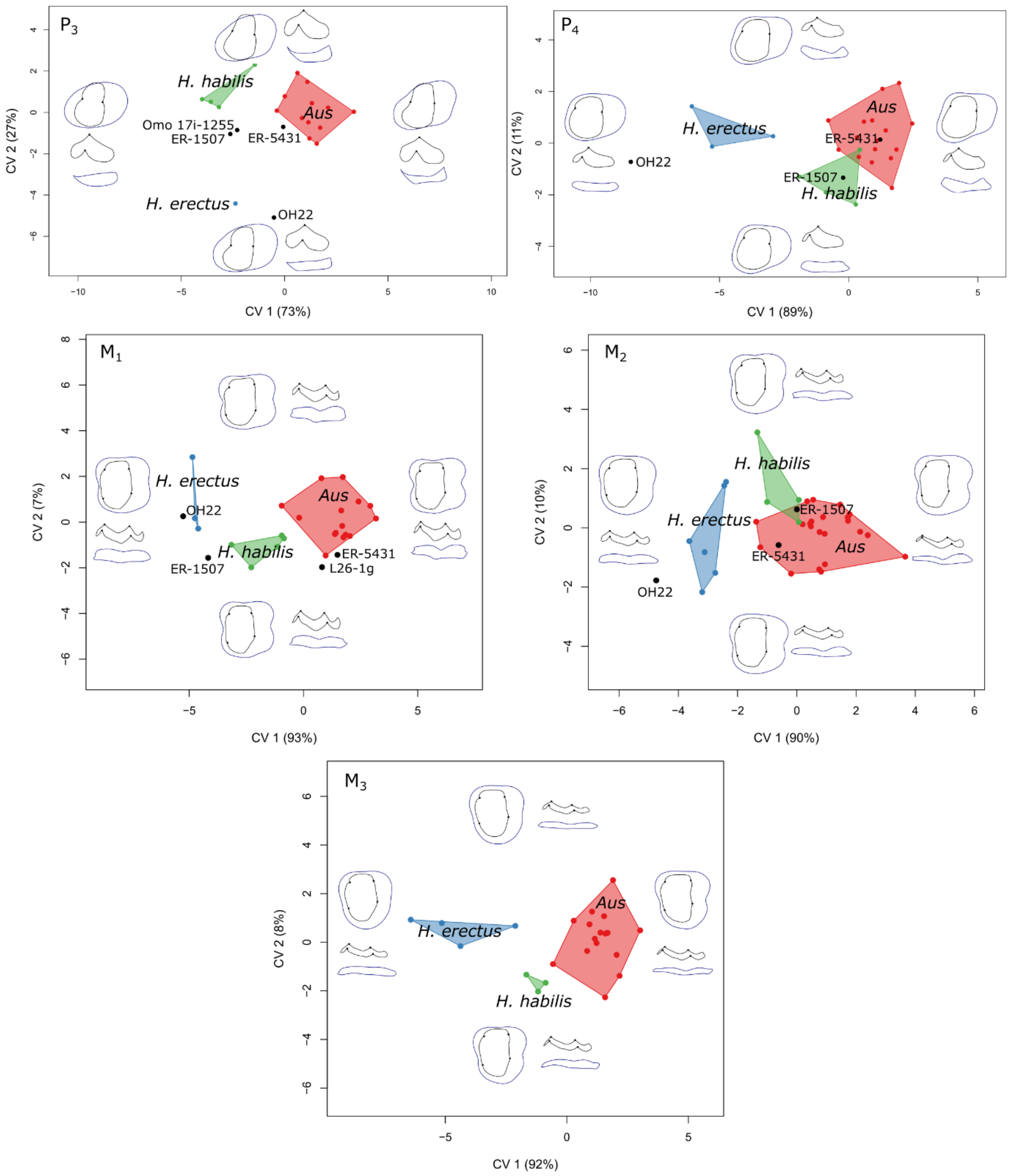

Supplementary Figure 13 – Canonical variate analysis (CVA) plots of EDJ shape in mandibular postcanine tooth positions. Wireframe images of CV (canonical variate) extremes are also shown (±1.5 standard deviations from the mean). *Aus* = *Australopithecus*. Source data are provided as a Source Data file.

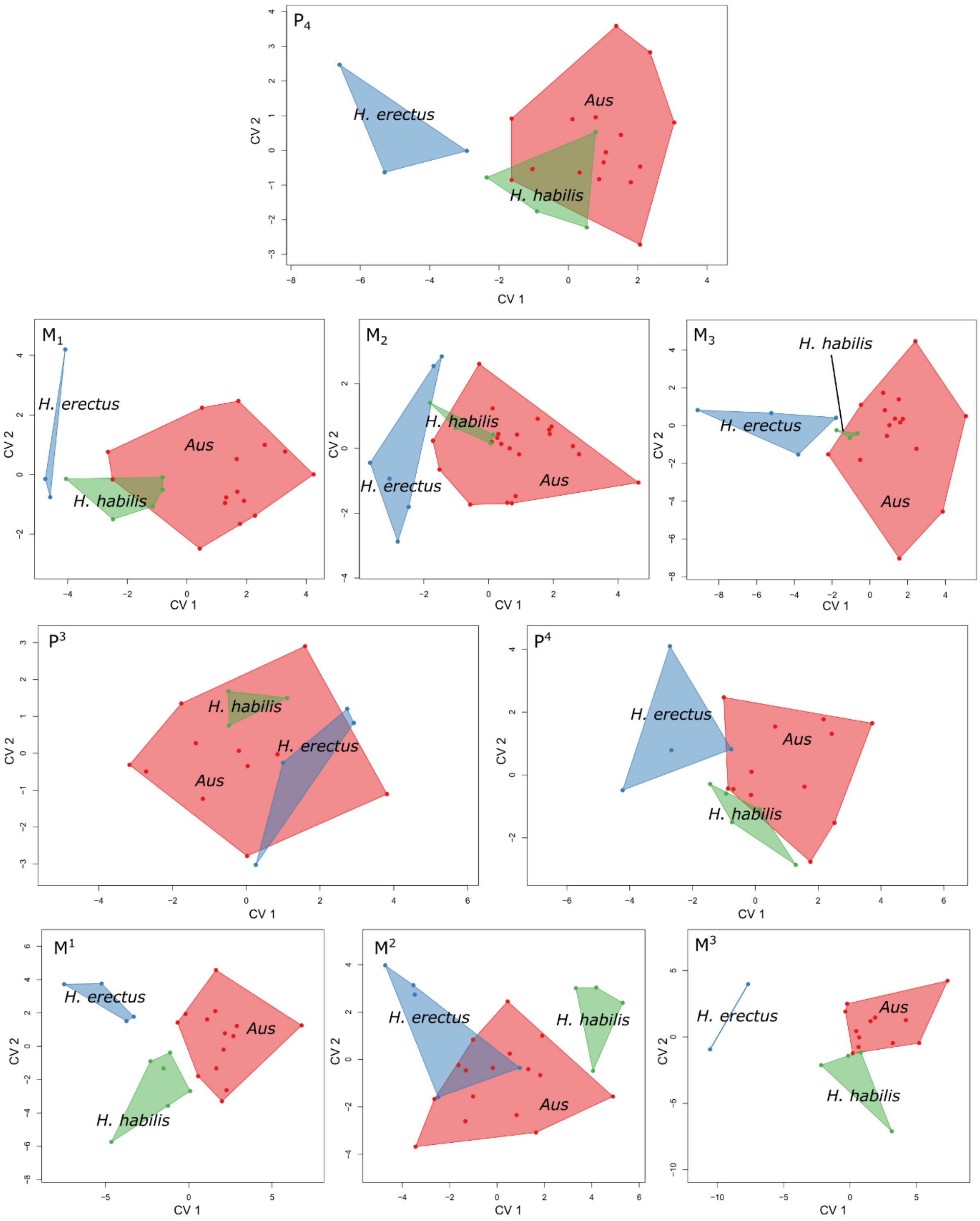

Supplementary Figure 14 – Cross-validated canonical variate analysis (cvCVA) plots of EDJ shape. *Aus* = *Australopithecus*. CV = canonical variate. Source data are provided as a Source Data file.

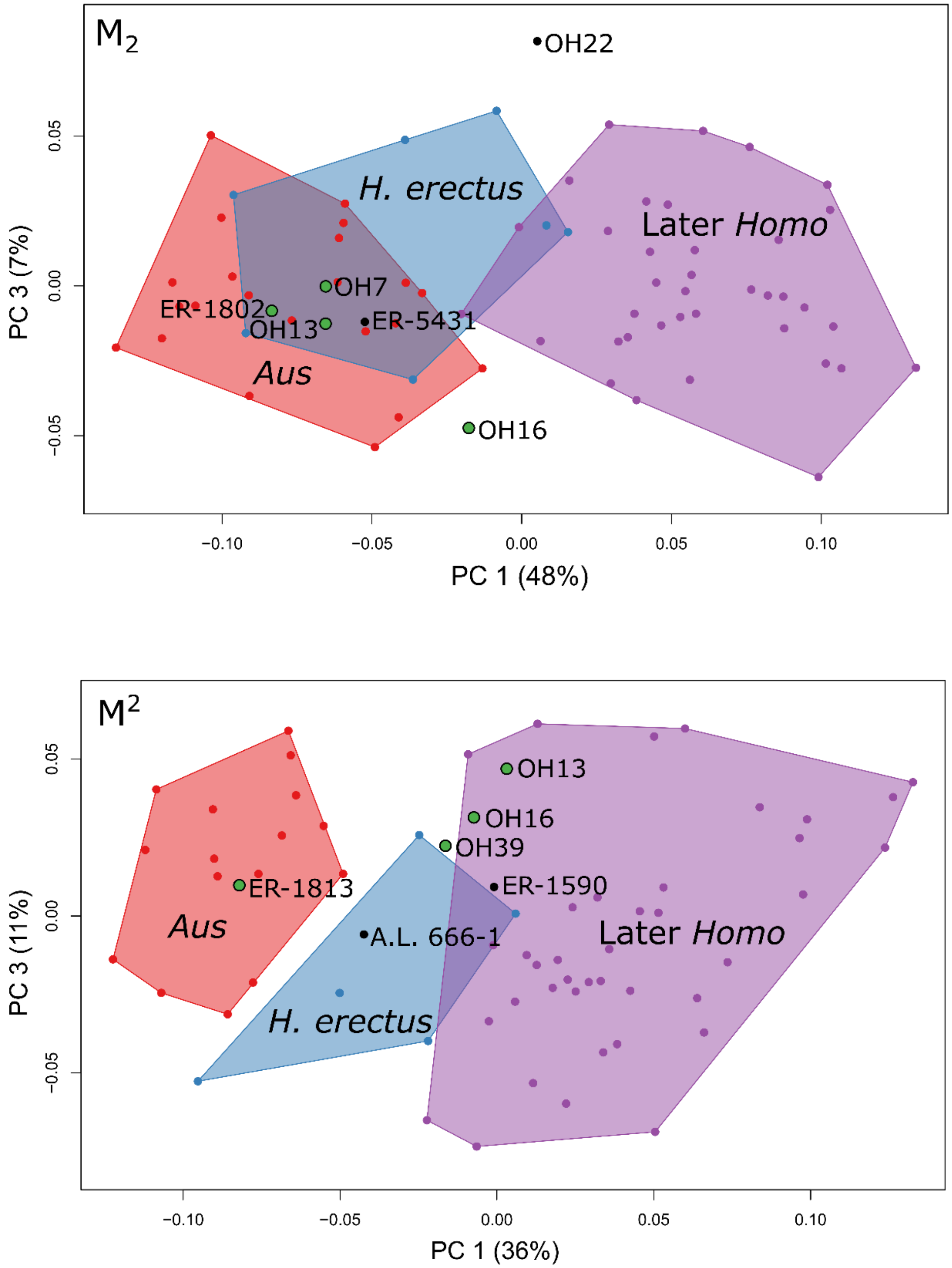

Supplementary Figure 15 – PCA plot showing variation in mandibular (top) and maxillary (bottom) second molar EDJ shape (Principal component 1 v 3). *Homo habilis* specimens are represented by green points. *Aus* = *Australopithecus*. Source data are provided as a Source Data file.

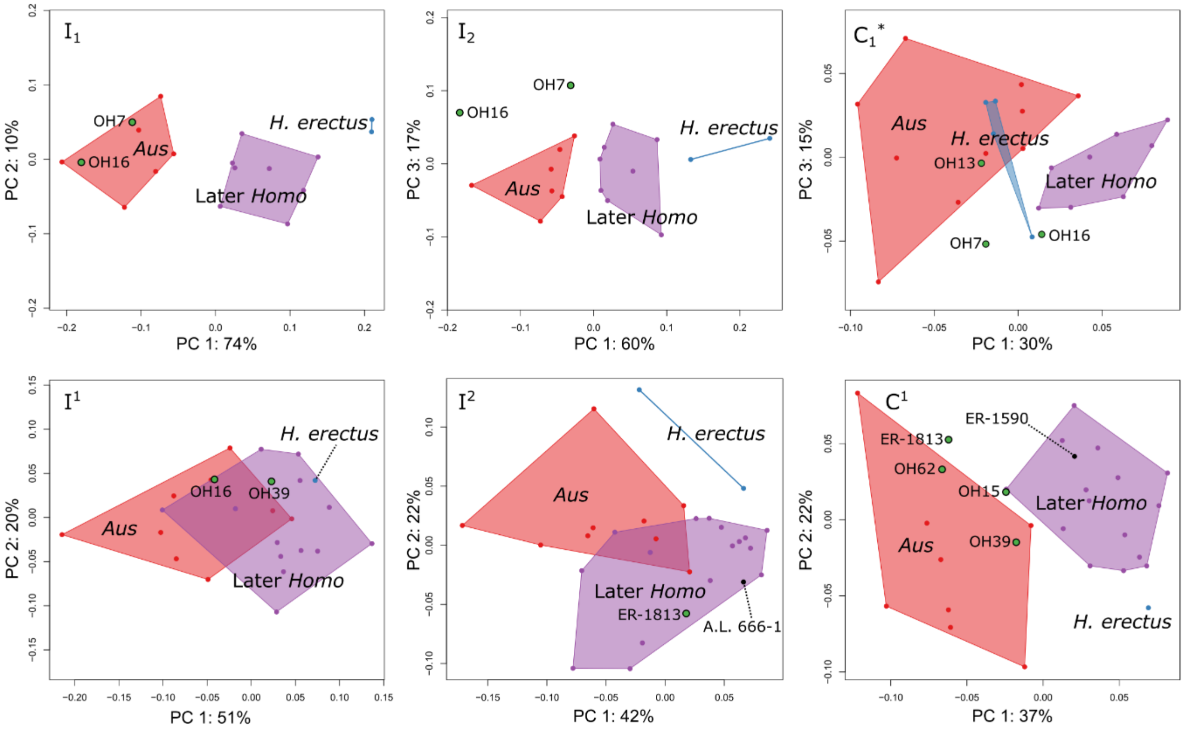

Supplementary Figure 16 – PCA plots showing variation in CEJ shape for each anterior tooth position. *Homo habilis* specimens are represented by green points. All plots show the first two principal components, except those marked with an asterisk (*), which show the first and third principal components. *Aus* = *Australopithecus*. Source Data are provided as a Source Data file.

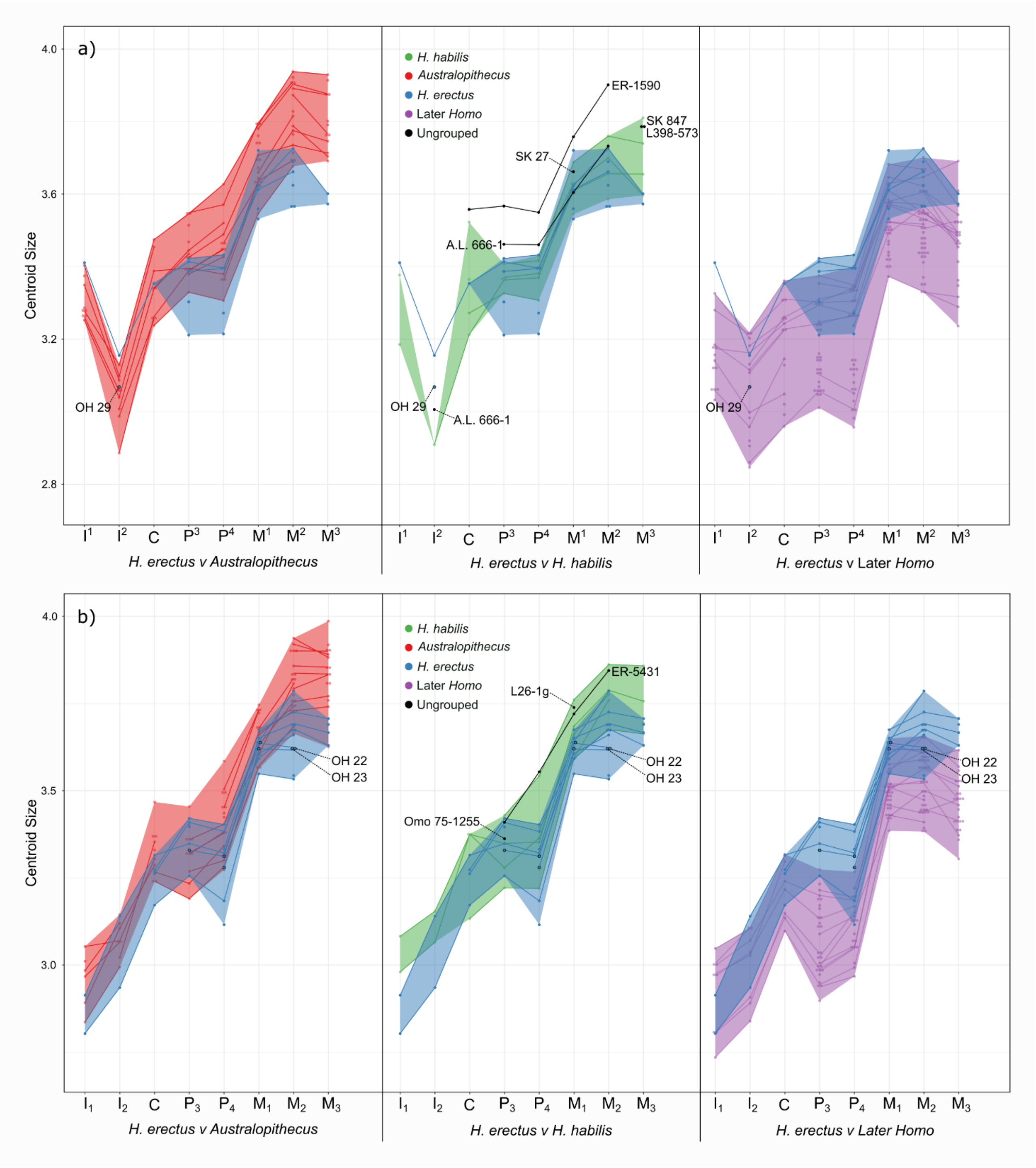

Supplementary Figure 17 – Centroid size across the tooth row – *Homo habilis* v *Homo erectus*. Plots show the natural logarithm of centroid size of the CEJ ridge landmark set, calculated separately for A) the mandibular tooth row, and B) the maxillary tooth row. *Australopithecus* is shown in red, later *Homo* in purple, and *Homo habilis* in green. Shaded areas represent the range of centroid sizes for each group, and lines connect teeth from the same individual. Specimens not attributed to a group are shown in black. Source data are available in Supplementary Data 21.

Supplementary Tables

Supplementary Table 1 – Shape indices (buccolingual/mesiodistal) for all currently known *Australopithecus afarensis, Australopithecus africanus* and *Homo habilis* (including A.L. 666-1) postcanine teeth, and results of two sided t-tests comparing *H. habilis* to a pooled *Australopithecus* sample. Source data are published measurements – see Supplementary Note 1 for more information.

		P^3	P^4	M^1	M^2	M^3	P_4	M_1	M_2	M_3
H. habilis	*n*	8	8	13	8	7	5	6	6	6
	Mean	132	130	100	110	115	103	86	89	85
	Range	124-145	125-140	93-108	104-122	105-125	100-109	83-90	85-93	81-90
	SD	8	5	4	7	7	3.7	2.7	2.9	2.9
A. afarensis	*n*	12	17	13	13	15	30	32	37	29
	Mean	142	138	110	113	115	112	94	95	88
	Range	136-151	123-151	96-121	110-119	106-123	80-135	85-103	85-108	81-101
	SD	5	7	7	3	5	11.3	4.2	5.6	4.5
A. africanus	*n*	24	21	25	27	29	25	29	39	34
	Mean	138	139	109	111	114	115	95	93	91
	Range	113-156	122-152	101-124	98-132	102-123	100-138	82-118	84-116	82-104
	SD	9	9	6	7	5	8.5	7.0	5.1	4.7
Australopithecus (pooled)	*n*	36	38	38	40	44	55	61	76	63
	Mean	139	139	110	112	114	113	95	94	90
	Range	113-156	122-152	96-124	98-132	102-123	80-138	82-118	84-116	81-104
	SD	8	8	6	6	5	10.1	5.6	5.5	4.8
	t-test v *H. habilis*	0.043*	0.0091**	0.000015***	0.53	0.78	0.026*	0.00065***	0.047*	0.028*

* = P ≤ 0.05, ** = P ≤ 0.01, *** = P ≤ 0.001. *p*-values are corrected for false discovery rate using Benjamini-Hochberg procedure

Supplementary Table 2 – Shape indices (buccolingual/mesiodistal) for a subset of *Australopithecus* and *Homo habilis* postcanine teeth that matches the specimens included in the EDJ analysis (Supplementary Table 3), and results of two sided t-tests comparing *H. habilis* to a pooled *Australopithecus* sample. Source data are published measurements – see Supplementary Note 1 for more information.

		P^3	P^4	M^1	M^2	M^3	P_4	M_1	M_2	M_3
H. habilis	*n*	4	6	7	5	4	4	5	4	4
	Mean	128	130	101	113	117	101	86	88	85
	Range	124-136	125-140	93-108	106-122	109-125	100-102	83-90	85-93	83-90
	SD	5.61	6.14	5.19	7.14	6.80	0.9	2.9	3.4	3.2
A. afarensis	*n*	4	6	4	4	3	8	7	9	3
	Mean	140	137	110	111	111	116	97	98	88
	Range	138-141	123-146	103-118	110-111	106-114	107-130	93-103	91-108	86-91
	SD	1.00	8.49	6.35	0.75	4.71	9.2	3.4	5.8	3.0
A. africanus	*n*	7	6	11	12	9	8	7	13	13
	Mean	144	140	106	111	115	109	91	91	91
	Range	134-156	122-152	101-113	105-119	110-120	100-117	86-95	86-96	83-98
	SD	7.45	11.76	4.41	3.98	4.35	5.5	2.5	2.9	4.6
Australopithecus (pooled)	*n*	11	12	15	16	12	16	14	22	16
	Mean	143	138	108	111	114	113	94	94	90
	Range	134-156	122-152	101-118	105-119	106-120	100-130	86-103	86-108	83-98
	SD	6.20	9.94	5.08	3.43	4.51	8.2	4.2	5.3	4.4
	t-test v *H. habilis*	0.019**	0.099	0.0086**	0.53	0.50	0.000068***	0.0011**	0.047*	0.031*

* = P ≤ 0.05, ** = P ≤ 0.01, *** = P ≤ 0.001. *p*-values are corrected for false discovery rate using Benjamini-Hochberg procedure

Supplementary Table 3 – *Homo habilis* dental hypodigm – specimens preserving whole tooth crowns. The accession number and fossil site is given for each specimen, as well as references supporting the attribution to *H. habilis*, and whether the specimen is included in the present sample.

Accession	Site	Attribution refs.	Included in sample?
KNM-ER 1502	Koobi Fora, Kenya	4	Yes
KNM-ER 1802	Koobi Fora, Kenya	34, 35	Yes
KNM-ER 1805	Koobi Fora, Kenya	4	No – poor tissue distinction in scan
KNM-ER 1813	Koobi Fora, Kenya	4	Yes
OH 4	Olduvai Gorge, Tanzania	1, 3	Yes
OH 6	Olduvai Gorge, Tanzania	1, 3	No - Only P_3 germ scanned, no cervix
OH 7	Olduvai Gorge, Tanzania	1, 3	Yes
OH 13	Olduvai Gorge, Tanzania	1, 3	Yes
OH 15	Olduvai Gorge, Tanzania	3	Yes
OH 16	Olduvai Gorge, Tanzania	1, 3	Yes
OH 21	Olduvai Gorge, Tanzania	3	Yes
OH 24	Olduvai Gorge, Tanzania	1, 3	Yes
OH 27	Olduvai Gorge, Tanzania	3	No – cervix incomplete
OH 37	Olduvai Gorge, Tanzania	3	Yes
OH 39	Olduvai Gorge, Tanzania	3	Yes
OH 41	Olduvai Gorge, Tanzania	3	No – not scanned
OH 44	Olduvai Gorge, Tanzania	3	No – cervix incomplete
OH 45	Olduvai Gorge, Tanzania	3	Yes
OH 62	Olduvai Gorge, Tanzania	3, 36	Yes
Omo L894-1	Omo, Ethiopia	37	No – no tissue distinction in scan

Supplementary Table 4. EDJ shape differences between *Australopithecus* and later *Homo*, and the condition observed in *Australopithecus* (*A. afarensis* v *A. africanus*), Koobi Fora *H. habilis* specimens, and A.L. 666-1. For more information on these features, see Table 2 of the main text.

Shape characteristics in later *Homo*	*A. afarensis v A. africanus*	KNM-ER 1802	KNM-ER 1813	A.L. 666-1
Increased relative dentine body height (all teeth)	No difference (P_3-M_3, P^3, M^1, M^3), *A. afarensis* slightly taller (P^3), or *A. africanus* slightly taller (M^2)	Dentine body short (P_4, M_2) or intermediate (P_3, M_1)	Dentine body short (P^4, M^1, M^2) or tall (M^3)	Dentine body intermediate (P^3, P^4, M^2)
Talon/talonid reduction (P_3, P_4, P^3, P^4)	No difference (P_3, P^3, P^4), or slight reduction in *A. africanus* (P_4)	No talonid reduction (P_3, P_4)	Moderate talon reduction (P^4)	No talonid reduction (P^3) or moderate lingual talonid reduction (P^4)
Metaconid reduced relative to protoconid (P_3, P_4)	No difference (P_3, P_4)	Metaconid slightly reduced in P_4, not in P_3	-	-
Protocone relatively taller and more distally placed (M^1, M^2, M^3) *	No difference (M^3), slightly taller in *A. africanus* (M^2) or slightly taller and more mesial in *A. africanus* (M^1)	-	Intermediate protocone height and placement (M^1), *Australopithecus*-like height and placement (M^2), or intermediate placement but height same as *Australopithecus* (M^3)	Intermediate protocone height and placement (M^2)
Reduced distal marginal ridge (DMR) (M^1, M^2, M^3)	DMR slightly reduced in *A. afarensis* (M^1, M^2, M^3)	-	DMR moderately reduced (M^2,M^3), or reduced only in distolingual component (M^1)	Moderate DMR reduction (M^2)
Protoconid relatively taller and more distally placed (M_1, M_2, M_3) *	Protoconid slightly taller in *A. africanus* (M_1, M_2), or slightly taller and distally placed (M_3)	Protoconid not taller or distally placed (M_1, M_2)	-	-
Rounded crown – EDJ ridge mesiodistally shorter and buccolingually wider (M_1, M_2, M_3) **	No difference (M_1, M_2), or *A. afarensis* less rounded	Less rounded crown, mesiodistally elongated (M_1, M_2)	-	-

Supplementary Table 5. EDJ shape differences between *Australopithecus* and later *Homo*, and the condition observed in *H. erectus* specimens. For more information, see Table 2 of the main text.

**Shape characteristics in later *Homo***	**KNM-ER 806**	**KNM-ER 992**	**KNM-ER 1507**	**OH 22**
Increased relative dentine body height (all teeth)	Dentine body short (M_1) or intermediate (M_2, M_3)	Dentine body intermediate (P_3, P_4, M_1, M_2, M_3)	Dentine body short (M_1, M_2) or intermediate (P_3,P_4)	Intermediate (P_3, P_4, M_2) or short (M_1) dentine body height
Talon/talonid reduction (P_3, P_4, P^3, P^4)	-	Talonid moderately reduced (P_3, P_4)	No talonid reduction (P_3, P_4)	Talonid reduced (P_3, P_4)
Metaconid reduced relative to protoconid (P_3, P_4)	-	Metaconid reduced (P_3) or slightly reduced (P_4)	Slight metaconid reduction (P_3, P_4)	Metaconid reduced (P_3,P_4)
Protoconid relatively taller and more distally placed (M_1, M_2, M_3) *	Protoconid variable (M_1 distally placed but short, M_2 neither tall nor distal, M_3 slightly taller and distal)	Protoconid variable (M_1 not taller or distal, M_2 taller and distal, M_3 slightly taller and distal)	Protoconid placement and height intermediate (M_1), or not tall or distal (M_2)	Protoconid taller and distally placed (M_1, M_2)
Rounded crown – EDJ ridge mesiodistally shorter and buccolingually wider (M_1, M_2, M_3) **	M_1 not rounded, M_2 rounded, M_3 slightly rounded	M_2 and M_3 rounded, M_1 not	Less rounded crown, mesiodistally elongated (M_1, M_2)	M_2 rounded, M_1 not

Supplementary Table 6 – Permutation test for shape and size differences between groups (two-sided). Procrustes coordinates from the full EDJ analysis are used in permutation tests. Bold indicates $p < 0.05$

Shape (Procrustes coordinates)						
**Tooth**	***Australopithecus* v later *Homo***	***H. habilis* v *Australopithecus***	***H. habilis* v *H. erectus***	***H. habilis* v later *Homo***	***H. erectus* v *Australopithecus***	***H. erectus* v later *Homo***
P^3	**0.0003**	0.0819	0.1087	**0.0018**	**0.0208**	**0.0003**
P^4	**0.0003**	0.1047	0.1180	**0.0003**	**0.0221**	**0.0003**
M^1	**0.0003**	0.1557	**0.0064**	**0.0003**	**0.0003**	**0.0003**
M^2	**0.0003**	**0.0030**	0.0703	**0.0120**	**0.0007**	**0.0049**
M^3	**0.0003**	**0.0074**	0.0911	**0.0007**	**0.0324**	**0.0251**
P_3	**0.0003**	**0.0039**	0.4113	**0.0003**	0.1769	0.0645
P_4	**0.0003**	0.1085	0.3045	**0.0007**	**0.0283**	**0.0021**
M_1	**0.0003**	0.1769	0.7143	**0.0003**	0.1959	**0.0019**
M_2	**0.0003**	0.3662	0.1375	**0.0003**	**0.0003**	**0.0003**
M_3	**0.0003**	0.1769	0.8004	**0.0005**	0.1029	**0.0014**

Size (natural logarithm of centroid size)						
**Tooth**	***Australopithecus* v later *Homo***	***H. habilis* v *Australopithecus***	***H. habilis* v *H. erectus***	***H. habilis* v later *Homo***	***H. erectus* v *Australopithecus***	***H. erectus* v later *Homo***
I^1	**0.0500**	0.9999	0.7803	0.0801	0.2103	0.1713
I^2	0.7662	0.3581	0.9999	0.4716	0.3581	0.8545
C	**0.0500**	0.9674	0.9999	0.3550	0.8587	0.5431
P^3	**0.0008**	0.2920	0.7803	**0.0079**	0.2920	**0.0021**
P^4	**0.0008**	0.3529	0.9999	**0.0256**	0.4716	**0.0210**
M^1	**0.0008**	0.2782	0.8598	**0.0438**	0.3040	0.0773
M^2	**0.0008**	0.1349	0.7344	**0.0127**	0.0601	**0.0261**
M^3	**0.0008**	0.3114	0.4480	**0.0108**	**0.0384**	0.3522
I_1	0.9999	0.6307	0.5360	0.6818	0.2920	0.5431
I_2	0.6802	0.6186	0.7803	0.5379	0.7803	0.9999
C	**0.0311**	0.6294	0.4656	0.1844	0.4861	0.7326
P_3	**0.0008**	0.7803	0.6294	**0.0019**	0.6473	**0.0008**
P_4	**0.0008**	0.5979	0.6818	**0.0048**	0.2092	**0.0025**
M_1	**0.0008**	0.9211	0.8057	**0.0021**	0.5431	**0.0073**
M_2	**0.0008**	0.8348	0.1844	**0.0019**	**0.0079**	**0.0008**
M_3	**0.0008**	0.0801	0.4308	**0.0019**	**0.0384**	**0.0021**

Supplementary Table 7 – Classification of unclassified specimens using CVA typicality probabilities. CVA analyses are conducted on subsets of principal components (between 5 and 10, resulting in 6 CVAs), with the specimens of interest projected a posteriori. Typicality probabilities are then calculated for each CVA, and specimens classified according to the highest typicality probability. When all typicality probabilities are below 0.1, specimens are not classified to any of the groups.

	P^3				P^4				M^1				M^2				M^3			
	Aus	*H. ere*	*H. hab*	NC	*Aus*	*H. ere*	*H. hab*	NC	*Aus*	*H. ere*	*H. hab*	NC	*Aus*	*H. ere*	*H. hab*	NC	*Aus*	*H. ere*	*H. hab*	NC
A.L. 666-1	6/6	0/6	0/6	0/6	6/6	0/6	0/6	0/6	-	-	-	-	2/6	0/6	1/6	3/6	-	-	-	-
KNM-ER 1590	6/6	0/6	0/6	0/6	6/6	0/6	0/6	0/6	0/6	0/6	2/6	4/6	0/6	1/6	0/6	5/6	-	-	-	-
SK 27	-	-	-	-	-	-	-	-	0/6	4/6	0/6	2/6	-	-	-	-	-	-	-	-
SK 847	-	-	-	-	-	-	-	-	-	-	-	-	-	-	-	-	0/6	1/6	0/6	5/6
L398-573	-	-	-	-	-	-	-	-	-	-	-	-	-	-	-	-	0/6	1/6	0/6	5/6

	P_3				P_4				M_1				M_2				M_3			
	Aus	*H. ere*	*H. hab*	NC	*Aus*	*H. ere*	*H. hab*	NC	*Aus*	*H. ere*	*H. hab*	NC	*Aus*	*H. ere*	*H. hab*	NC	*Aus*	*H. ere*	*H. hab*	NC
KNM-ER 1507	0/6	0/6	4/6	2/6	5/6	0/6	1/6	0/6	0/6	1/6	3/6	2/6	1/6	0/6	5/6	0/6	-	-	-	-
KNM-ER 5431	6/6	0/6	0/6	0/6	6/6	0/6	0/6	0/6	6/6	0/6	0/6	0/6	6/6	0/6	0/6	0/6	-	-	-	-
OH 22	0/6	4/6	0/6	2/6	0/6	0/6	0/6	6/6	0/6	5/6	0/6	1/6	0/6	1/6	0/6	5/6	-	-	-	-
Omo 75i-1255	0/6	0/6	4/6	2/6	-	-	-	-	-	-	-	-	-	-	-	-	-	-	-	-
L26-1g	-	-	-	-	-	-	-	-	1/6	0/6	0/6	5/6	-	-	-	-	-	-	-	-

Abbreviations: *Aus* = *Australopithecus*, *H. ere* = *H. erectus*, *H. hab* = *H. habilis*, NC = Not classified

Supplementary References

1. Leakey, L. S. B., Tobias, P. V. & Napier, J. R. A New Species of The Genus *Homo* From Olduvai Gorge. *Nature* **202**, 7–9 (1964).
2. Tobias, P. V. The Distinctiveness of *Homo habilis*. *Nature* vol. 209 953–957 (1966).
3. Tobias, P. V. *The Skulls, Endocasts, and Teeth of* Homo habilis*: pt. 1-4*. (Cambridge University Press, 1991).
4. Wood, B. A. *Koobi Fora Research Project: Volume 4. Hominid cranial remains*. *Koobi Fora Research Project* (Clarendon Press, 1991).
5. White, T. D., Johanson, D. C. & Kimbel, W. H. *Australopithecus africanus*: Its Phyletic Position Reconsidered. in *New Interpretations of Ape and Human Ancestry* (eds. Ciochon, R. L. & Corruccini, R. S.) 721–780 (Springer, 1983).
6. Suwa, G., White, T. D. & Howell, F. C. Mandibular postcanine dentition from the Shungura Formation, Ethiopia: Crown morphology, taxonomic allocations, and Plio-Pleistocene hominid evolution. *American Journal of Physical Anthropology* vol. 101 247–282 (1996).
7. Kimbel, W. H., Johanson, D. C. & Rak, Y. Systematic assessment of a maxilla of *Homo* from Hadar, Ethiopia. *American Journal of Physical Anthropology: The Official Publication of the American Association of Physical Anthropologists* **103**, 235–262 (1997).
8. White, T. D. New fossil hominids from Laetolil, Tanzania. *American Journal of Physical Anthropology* **46**, 197–229 (1977).
9. White, T. D. Additional fossil hominids from Laetoli, Tanzania: 1976–1979 specimens. *American Journal of Physical Anthropology* **53**, 487–504 (1980).
10. Kimbel, W. H., Rak, Y. & Johanson, D. C. *The skull of* Australopithecus afarensis. (Oxford University Press, 2004).
11. White, T. D., Suwa, G., Simpson, S. & Asfaw, B. Jaws and teeth of *Australopithecus afarensis* from Maka, Middle Awash, Ethiopia. *American Journal of Physical Anthropology* **111**, 45–68 (2000).
12. Harrison, T. Hominins from the Upper Laetolil and Upper Ndolanya Beds, Laetoli. in *Paleontology and Geology of Laetoli: Human Evolution in Context* 141–188 (Springer, 2011).
13. Haile-Selassie, Y. *et al.* Dentognathic remains of *Australopithecus afarensis* from Nefuraytu (Woranso-Mille, Ethiopia): Comparative description, geology, and paleoecological context. *Journal of Human Evolution* **100**, 35–53 (2016).
14. Clarke, R. J. & Kuman, K. The skull of StW 573, a 3.67 ma *Australopithecus prometheus* skeleton from Sterkfontein Caves, South Africa. *Journal of Human Evolution* **134**, 102634 (2019).
15. Haile-Selassie, Y. & Ryan, T. M. Comparative description and taxonomy of new hominin juvenile mandibles from the Pliocene of Woranso-Mille (Central Afar, Ethiopia). *Journal of Human Evolution* **132**, 15–31 (2019).
16. Melillo, S. M. *et al.* New Pliocene hominin remains from the Leado Dido'a area of Woranso-Mille, Ethiopia. *Journal of Human Evolution* **153**, 102956 (2021).

17. Moggi-Cecchi, J., Grine, F.E. and Tobias, P.V., Early hominid dental remains from Members 4 and 5 of the Sterkfontein Formation (1966–1996 excavations): catalogue, individual associations, morphological descriptions and initial metrical analysis. *Journal of Human Evolution*, **50,** 239–328 (2006).

18. Beynon, A. D. & Wood, B. A. Variations in enamel thickness and structure in East African hominids. *American Journal of Physical Anthropology* **70**, 177–193 (1986).

19. White, T. D., Suwa, G. & Asfaw, B. *Australopithecus ramidus*, a new species of early hominid from Aramis, Ethiopia. *Nature* **371**, 306–312 (1994).

20. Grine, F. E. & Martin, L. Enamel thickness and development in *Australopithecus* and *Paranthropus*. in *Evolutionary History of the Robust Australopithecines* (ed. Grine, F. E.) 3–42 (Aldine de Gruyter, 1988).

21. Schwartz, G. T. Taxonomic and functional aspects of the patterning of enamel thickness distribution in extant large-bodied hominoids. *American Journal of Physical Anthropology: The Official Publication of the American Association of Physical Anthropologists* **111**, 221–244 (2000).

22. Schwartz, G. T. *Taxonomic and functional aspects of enamel cap structure in South Africa Plio-Pleistocene hominids: A high-resolution computed tomographic study*. (Washington University in St. Louis, 1997).

23. Olejniczak, A. J. *et al.* Dental tissue proportions and enamel thickness in Neandertal and modern human molars. *Journal of human evolution* **55**, 12–23 (2008).

24. Smith, T. M. *et al.* Variation in enamel thickness within the genus *Homo*. *Journal of human evolution* **62**, 395–411 (2012).

25. Skinner, M. M., Alemseged, Z., Gaunitz, C. & Hublin, J.-J. Enamel thickness trends in Plio-Pleistocene hominin mandibular molars. *Journal of human evolution* vol. 85 35–45 (2015).

26. Lockey, A. L., Alemseged, Z., Hublin, J.-J. & Skinner, M. M. Maxillary molar enamel thickness of Plio-Pleistocene hominins. *Journal of Human Evolution* **142**, 102731 (2020).

27. Kono, R. T., Suwa, G. & Tanijiri, T. A three-dimensional analysis of enamel distribution patterns in human permanent first molars. *Archives of Oral Biology* **47**, 867–875 (2002).

28. Kono, R. T. Molar enamel thickness and distribution patterns in extant great apes and humans: new insights based on a 3-dimensional whole crown perspective. *Anthropological Science* vol. 112 121–146 (2004).

29. Olejniczak, A. J. *et al.* Three-dimensional molar enamel distribution and thickness in *Australopithecus* and *Paranthropus*. *Biology Letters* **4**, 406–410 (2008).

30. Pan, L. *et al.* Further morphological evidence on South African earliest *Homo* lower postcanine dentition: Enamel thickness and enamel dentine junction. *Journal of human evolution* vol. 96 82–96 (2016).

31. Martín-Francés, L. *et al.* Crown tissue proportions and enamel thickness distribution in the Middle Pleistocene hominin molars from Sima de los Huesos (SH) population (Atapuerca, Spain). *PloS one* **15**, e0233281 (2020).

32. Suwa, G. *et al.* Paleobiological implications of the *Ardipithecus ramidus* dentition. *Science (New York, N.Y.)* **326**, 94–99 (2009).

33. Johanson, D. C., White, T. D. & Coppens, Y. Dental remains from the Hadar Formation, Ethiopia: 1974–1977 collections. *American Journal of Physical Anthropology* **57**, 545–603 (1982).

34. Leakey, R. E. F. Further evidence of Lower Pleistocene hominids from East Rudolf, North Kenya, 1973. *Nature* **248**, 653 (1974).

35. Spoor, F. *et al.* Reconstructed *Homo habilis* type OH 7 suggests deep-rooted species diversity in early Homo. *Nature* **519**, 83 (2015).

36. Johanson, D. C. *et al.* New partial skeleton of *Homo habilis* from Olduvai Gorge, Tanzania. *Nature* **327**, 205–209 (1987).

37. Boaz, N. T. & Howell, F. C. A gracile hominid cranium from Upper Member G of the Shungura Formation, Ethiopia. *American Journal of Physical Anthropology* **46**, 93–108 (1977).
