# Supplementary material for: Dental morphology in Homo habilis and its implications for the evolution of early Homo
Source: Nat Commun. 2024 Jan 4;15:286. doi: 10.1038/s41467-023-44375-9 (PMC10767101; doi:10.1038/s41467-023-44375-9)
Supplement: Supplementary file 3 — Description of Additional Supplementary Files [file 41467_2023_44375_MOESM3_ESM.pdf]

Source Data – This file contains the source data for Figures 3-5 and Supplementary Figures 7, 10-16.

Supplementary Data 1 – Interactive html PCA plot showing variation in P_3 EDJ shape. Each specimen's accession number and taxonomic attribution can be viewed by hovering with the mouse.

Supplementary Data 2 – Interactive html PCA plot showing variation in P_4 EDJ shape. Each specimen's accession number and taxonomic attribution can be viewed by hovering with the mouse.

Supplementary Data 3 – Interactive html PCA plot showing variation in M_1 EDJ shape. Each specimen's accession number and taxonomic attribution can be viewed by hovering with the mouse.

Supplementary Data 4 – Interactive html PCA plot showing variation in M_2 EDJ shape. Each specimen's accession number and taxonomic attribution can be viewed by hovering with the mouse.

Supplementary Data 5 – Interactive html PCA plot showing variation in M_3 EDJ shape. Each specimen's accession number and taxonomic attribution can be viewed by hovering with the mouse.

Supplementary Data 6 – Interactive html PCA plot showing variation in P^3 EDJ shape. Each specimen's accession number and taxonomic attribution can be viewed by hovering with the mouse.

Supplementary Data 7 – Interactive html PCA plot showing variation in P^4 EDJ shape. Each specimen's accession number and taxonomic attribution can be viewed by hovering with the mouse.

Supplementary Data 8 – Interactive html PCA plot showing variation in M^1 EDJ shape. Each specimen's accession number and taxonomic attribution can be viewed by hovering with the mouse.

Supplementary Data 9 – Interactive html PCA plot showing variation in M^2 EDJ shape. Each specimen's accession number and taxonomic attribution can be viewed by hovering with the mouse.

Supplementary Data 10 – Interactive html PCA plot showing variation in M^3 EDJ shape. Each specimen's accession number and taxonomic attribution can be viewed by hovering with the mouse.

Supplementary Data 11 – Interactive html plot showing centroid size across the maxillary tooth row in *Homo habilis* v *Australopithecus.* Each specimen's accession number and taxonomic attribution can be viewed by hovering with the mouse.

Supplementary Data 12 – Interactive html plot showing centroid size across the maxillary tooth row in *Homo habilis* v *Homo erectus.* Each specimen's accession number and taxonomic attribution can be viewed by hovering with the mouse.

Supplementary Data 13 – Interactive html plot showing centroid size across the maxillary tooth row in *Homo habilis* v later *Homo.* Each specimen's accession number and taxonomic attribution can be viewed by hovering with the mouse.

Supplementary Data 14 – Interactive html plot showing centroid size across the mandibular tooth row in *Homo habilis* v *Australopithecus.* Each specimen's accession number and taxonomic attribution can be viewed by hovering with the mouse.

Supplementary Data 15 – Interactive html plot showing centroid size across the mandibular tooth row in *Homo habilis* v *Homo erectus.* Each specimen's accession number and taxonomic attribution can be viewed by hovering with the mouse.

Supplementary Data 16 – Interactive html plot showing centroid size across the mandibular tooth row in *Homo habilis* v later *Homo.* Each specimen's accession number and taxonomic attribution can be viewed by hovering with the mouse.

Supplementary Data 17 – Interactive html plot showing centroid size across the maxillary tooth row in *Homo erectus* v *Australopithecus.* Each specimen's accession number and taxonomic attribution can be viewed by hovering with the mouse.

Supplementary Data 18 – Interactive html plot showing centroid size across the maxillary tooth row in *Homo erectus* v later *Homo*. Each specimen's accession number and taxonomic attribution can be viewed by hovering with the mouse.

Supplementary Data 19 – Interactive html plot showing centroid size across the mandibular tooth row in *Homo erectus* v *Australopithecus.* Each specimen's accession number and taxonomic attribution can be viewed by hovering with the mouse.

Supplementary Data 20 – Interactive html plot showing centroid size across the mandibular tooth row in *Homo erectus* v later *Homo*. Each specimen's accession number and taxonomic attribution can be viewed by hovering with the mouse.

Supplementary Data 21 – Full hominin study sample for geometric morphometric analyses with information on each specimen.

Supplementary Data 22 – Full extant ape study sample with information on each specimen.
