# Supplementary material for: Dental morphology in Homo habilis and its implications for the evolution of early Homo
Source: Nat Commun. 2024 Jan 4;15:286. doi: 10.1038/s41467-023-44375-9 (PMC10767101; doi:10.1038/s41467-023-44375-9)
Supplement: Supplementary file 5 — Reporting Summary [file 41467_2023_44375_MOESM5_ESM.pdf]

nature portfolio

Corresponding author(s): Thomas W Davies

Last updated by author(s): Dec 5, 2023

Reporting Summary

Nature Portfolio wishes to improve the reproducibility of the work that we publish. This form provides structure for consistency and transparency in reporting. For further information on Nature Portfolio policies, see our Editorial Policies and the Editorial Policy Checklist.

Statistics

For all statistical analyses, confirm that the following items are present in the figure legend, table legend, main text, or Methods section.

n/a	Confirmed	
☐	☒	The exact sample size (*n*) for each experimental group/condition, given as a discrete number and unit of measurement
☐	☒	A statement on whether measurements were taken from distinct samples or whether the same sample was measured repeatedly
☐	☒	The statistical test(s) used AND whether they are one- or two-sided<br>*Only common tests should be described solely by name; describe more complex techniques in the Methods section.*
☐	☒	A description of all covariates tested
☐	☒	A description of any assumptions or corrections, such as tests of normality and adjustment for multiple comparisons
☐	☒	A full description of the statistical parameters including central tendency (e.g. means) or other basic estimates (e.g. regression coefficient) AND variation (e.g. standard deviation) or associated estimates of uncertainty (e.g. confidence intervals)
☐	☒	For null hypothesis testing, the test statistic (e.g. *F*, *t*, *r*) with confidence intervals, effect sizes, degrees of freedom and *P* value noted<br>*Give P values as exact values whenever suitable.*
☒	☐	For Bayesian analysis, information on the choice of priors and Markov chain Monte Carlo settings
☒	☐	For hierarchical and complex designs, identification of the appropriate level for tests and full reporting of outcomes
☒	☐	Estimates of effect sizes (e.g. Cohen's *d*, Pearson's *r*), indicating how they were calculated

Our web collection on statistics for biologists contains articles on many of the points above.

Software and code

Policy information about availability of computer code

Data collection	MIA v.2.4.6, Avizo v.6.3, Avizo v.8.0, Geomagic Studio 2014
Data analysis	Morpho (v.2.1.1) and princurve (v2.1.6) for R (v.4.3.1) - R code used for geometric morphometric analysis is freely available from: https://zenodo.org/doi/10.5281/zenodo.10255288

For manuscripts utilizing custom algorithms or software that are central to the research but not yet described in published literature, software must be made available to editors and reviewers. We strongly encourage code deposition in a community repository (e.g. GitHub). See the Nature Portfolio guidelines for submitting code & software for further information.

Data

Policy information about availability of data

All manuscripts must include a data availability statement. This statement should provide the following information, where applicable:

- Accession codes, unique identifiers, or web links for publicly available datasets
- A description of any restrictions on data availability
- For clinical datasets or third party data, please ensure that the statement adheres to our policy

The geometric morphometric landmark data generated in this study have been deposited in the Publications section of The Human Fossil Record (https://human-fossil-record.org/index.php?/category/14230). The raw scan data used in this study are curated by the museums and institutes who curate the original fossil material. This data were used under a MOU for the current study, and so are not publicly available, but can be accessed by research application to the relevant

curatorial institution (see Supplementary Table 8). The source data for Figures 3, 4, 5, and Supplementary Figures 7, 10, 11, 12, 13, 14, 15 and 16 are provided as a Source Data file. The source data for Figure 6 and Supplementary Figure 17 are available in Supplementary Table 8. Supplementary Tables 1 and 2 are based on previously published data, which is available in references listed in Supplementary Note 1.

Research involving human participants, their data, or biological material

Policy information about studies with human participants or human data. See also policy information about sex, gender (identity/presentation), and sexual orientation and race, ethnicity and racism.

Reporting on sex and gender	*Use the terms sex (biological attribute) and gender (shaped by social and cultural circumstances) carefully in order to avoid confusing both terms. Indicate if findings apply to only one sex or gender; describe whether sex and gender were considered in study design; whether sex and/or gender was determined based on self-reporting or assigned and methods used. Provide in the source data disaggregated sex and gender data, where this information has been collected, and if consent has been obtained for sharing of individual-level data; provide overall numbers in this Reporting Summary. Please state if this information has not been collected. Report sex- and gender-based analyses where performed, justify reasons for lack of sex- and gender-based analysis.*
Reporting on race, ethnicity, or other socially relevant groupings	*Please specify the socially constructed or socially relevant categorization variable(s) used in your manuscript and explain why they were used. Please note that such variables should not be used as proxies for other socially constructed/relevant variables (for example, race or ethnicity should not be used as a proxy for socioeconomic status). Provide clear definitions of the relevant terms used, how they were provided (by the participants/respondents, the researchers, or third parties), and the method(s) used to classify people into the different categories (e.g. self-report, census or administrative data, social media data, etc.) Please provide details about how you controlled for confounding variables in your analyses.*
Population characteristics	*Describe the covariate-relevant population characteristics of the human research participants (e.g. age, genotypic information, past and current diagnosis and treatment categories). If you filled out the behavioural & social sciences study design questions and have nothing to add here, write "See above."*
Recruitment	*Describe how participants were recruited. Outline any potential self-selection bias or other biases that may be present and how these are likely to impact results.*
Ethics oversight	*Identify the organization(s) that approved the study protocol.*

Note that full information on the approval of the study protocol must also be provided in the manuscript.

Field-specific reporting

Please select the one below that is the best fit for your research. If you are not sure, read the appropriate sections before making your selection.

☐ Life sciences ☐ Behavioural & social sciences ☒ Ecological, evolutionary & environmental sciences

For a reference copy of the document with all sections, see nature.com/documents/nr-reporting-summary-flat.pdf

Ecological, evolutionary & environmental sciences study design

All studies must disclose on these points even when the disclosure is negative.

Study description	This study uses geometric morphometrics to quantitatively analyze the enamel-dentine junction (EDJ) morphology of Homo habilis, and comparing with a broad comparative hominin sample. Geometric morphometric (GM) analysis was conducted separately on each permanent tooth position, of the full EDJ for postcanine teeth, and the cervix shape for canines and incisors. Separate GM analyses were conducted for mandibular and maxillary teeth at the cervix, across tooth positions, to investigate size patterns across the tooth row. Finally, separate GM analyses were conducted of the EDJ morphology of postcanine teeth (P3-M2) in order to compare levels of variation in Homo habilis with extant apes and humans.
Research sample	The research sample consists of micro-CT scans of Homo habilis teeth, and a comparative sample of hominin teeth attributed to Australopithecus, early Homo, Homo erectus and later Homo. These samples were chosen as they provide the most appropriate comparison for Homo habilis. An additional sample of Pan troglodytes, Gorilla gorilla and Homo sapiens (also included in the later Homo sample above) teeth were included as a modern reference to contextualize the variation observed within the Homo habilis sample. Micro-CT data for specimens from Olduvai Gorge was collected for this study after acceptance of a research permit (#2017-182-NA-2016-304) to the Tanzanian Commission for Science and Technology (COSTECH) and under a memorandum of understanding signed between MMS and the National Museum of Tanzania. Micro-CT data for comparative specimens was collected in agreement with a range of curatorial institutions as listed in Supplementary Table 8.
Sampling strategy	Fossil samples were chosen to provide an appropriate context for Homo habilis. Sample sizes were determined by the availability of fossils, their preservation and the quality of the tissue contrast in micro-CT scans.
Data collection	F.S., Z.A., A.G., J.-J.H., W.K., M.M.S contributed to microCT scanning of fossils. Micro-CT scan data processing and landmark collection was performed by T.W.D, W.P., C.Z. and M.M.S.
Timing and spatial scale	Micro-CT scans were processed, and landmark data collected, between 2009 and 2023.

Data exclusions	Data was excluded where fossil preservation was not sufficient, for example if a tooth is overly worn, broken or has insufficient contrast between tissue types in CT scans. Antimeres were excluded, as well as teeth with developmental abnormalities.
Reproducibility	This study follows well-established GM and landmarking protocols, and as such no new reproducibility or observer error tests were undertaken.
Randomization	Specimens were allocated into groups based on previously published taxonomic attributions. This is discussed within the main text for several specimens with less-clear attributions.
Blinding	No blinding was used.

Did the study involve field work? ☐ Yes ☒ No

Reporting for specific materials, systems and methods

We require information from authors about some types of materials, experimental systems and methods used in many studies. Here, indicate whether each material, system or method listed is relevant to your study. If you are not sure if a list item applies to your research, read the appropriate section before selecting a response.

Materials & experimental systems

n/a	Involved in the study
☒	☐ Antibodies
☒	☐ Eukaryotic cell lines
☐	☒ Palaeontology and archaeology
☒	☐ Animals and other organisms
☒	☐ Clinical data
☒	☐ Dual use research of concern
☒	☐ Plants

Methods

n/a	Involved in the study
☒	☐ ChIP-seq
☒	☐ Flow cytometry
☒	☐ MRI-based neuroimaging

Palaeontology and Archaeology

Specimen provenance	The provenance of specimens is listed in Table S8 for hominins, and in Table S9 for extant apes. Scan data for specimens from Olduvai Gorge was collected for this study after acceptance of a research permit (#2017-182-NA-2016-304) to the Tanzanian Commission for Science and Technology (COSTECH) and under a memorandum of understanding signed between MMS and the National Museum of Tanzania. Comparative data was used under agreement with the below curatorial institutions.
Specimen deposition	Specimens are deposited in the following institutions: National Museum of Tanzania, National Museums of Kenya, Ethiopian Authority for Research and Conservation of Cultural heritage, Evolutionary Studies Institute - University of the Witwatersrand, Ditsong Museum, Croatian Natural History Museum, ASBL Archéologie Andennaise - Royal Belgian Institute of Natural Sciences, Musée National de Préhistoire des Eyzies-de-Tayac, Musée d'Art et d'Archéologie du Périgord, Institut für Anatomie der Universität Leipzig, Museo Nacional de Ciencias Naturales, Croatian Academy of Sciences and Arts, Musée d'Angoulême, Musée d'Archéologie nationale de Saint-Germain-en-Laye, National Museum of Natural History, Institut für Geowissenschaften - Universität Heidelberg, Department of Archaeology - Belgrade University and the National Museum, Ephorate of Palaeoanthropology & Speleology of Southern Greece, Phyletisches Museum Jena, Senckenberg Forschungsinstitut und Naturmuseum - Frankfurt, Museum für Naturkunde - Leibniz Institute for Evolution and Biodiversity Science, Royal Museum for Central Africa - Tervuren, Max Planck Institute for Evolutionary Anthropology, and the Senckenberg, Forschungsstation für Quartärpaläontologie
Dating methods	No new dates are provided in this paper

☐ Tick this box to confirm that the raw and calibrated dates are available in the paper or in Supplementary Information.

Ethics oversight	Ethical guidance on the use of fossil specimens follows that set out by the relevant curatorial institutions.

Note that full information on the approval of the study protocol must also be provided in the manuscript.
